# Heated tobacco product-derived aerosol extract impairs gingival epithelial barrier function via CXADR downregulation

**DOI:** 10.1016/j.toxrep.2026.102346

**Published:** 2026-09-25

**Authors:** Shunsuke Yamaga, Keita Tanigaki, Akito Sakanaka, Atsuo Amano, Michiya Matsusaki, Hiroki Takeuchi, Masae Kuboniwa

**Affiliations:** a Department of Preventive Dentistry, The Osaka University Dental Hospital, Suita, Osaka, 565-0871, Japan; b Department of Preventive Dentistry, Graduate School of Dentistry, The Osaka University, Suita, Osaka, 565-0871, Japan; c Joint Research Laboratory (TOPPAN) for Advanced Cell Regulatory Chemistry, Graduate School of Engineering, The Osaka University, Suita, Osaka, 565-0871, Japan; d Department of Applied Chemistry, Graduate School of Engineering, The Osaka University, Suita, Osaka, 565-0871, Japan

**Keywords:** Periodontitis, CXADR, NFE2L1, Heated tobacco product, Tobacco, LPS, Epithelial, cell, Permeability

## Abstract

In our previous study, coxsackievirus and adenovirus receptor (CXADR), a tight junction-associated protein, was shown to play a critical role in maintaining gingival epithelial barrier function. Smoking is a well-established risk factor for periodontal disease; however, the effects of heated tobacco products on epithelial barrier function remain poorly understood. The present study investigated the effects of three heated tobacco product extracts (HTPE) on CXADR expression in human gingival epithelial cells. Exposure to the three HTPE significantly reduced CXADR expression, and functional analyses supported the involvement of nuclear factor erythroid 2-like 1 (NFE2L1), a CNC-bZIP transcription factor, in the three HTPE-induced CXADR downregulation in gingival epithelial cells. In a three-dimensional multilayered gingival epithelial tissue model, HTPE exposure increased permeability to 40 kDa dextran and lipopolysaccharide, whereas CXADR overexpression attenuated the three HTPE-induced increase in permeability to these substrates. These findings indicate that the three HTPE compromise gingival barrier integrity, with CXADR downregulation contributing to increased permeability to bacterial components.

## Highlights

• Heated tobacco product-derived aerosol extract (HTPE) decreases NFE2L1 expression in gingival epithelial cells.
• Decrease of NFE2L1 by HTPE causes loss of CXADR, a tight junction-related protein.
• HTPE dampens barrier function of gingival epithelial tissues.

## Graphical Abstract

**Figure:**
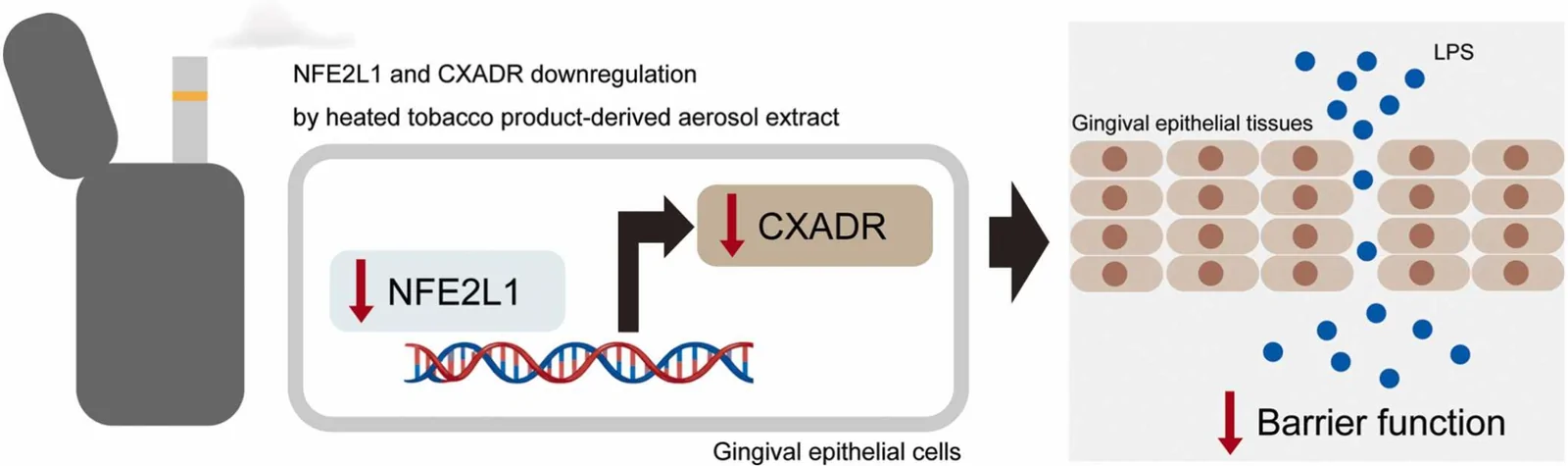

## 1 Introduction

Periodontitis is a chronic inflammatory disease driven by dysregulated host immune responses to dysbiotic microbial communities, leading to the progressive destruction of periodontal supporting tissues. [1] (Lamont et al., 2023, FEMS Microbiol Rev) That same study further demonstrated that tissue destruction is mediated by host-derived molecular and cellular mechanisms, including excessive inflammatory signaling, oxidative stress, and imbalanced bone remodeling. Importantly, environmental exposures that modulate these pathways can critically influence susceptibility to disease and progression.

Tobacco exposure is a major environmental modifier of periodontal disease pathogenesis. [2] (Apatzidou DA., 2022, Periodontol 2000) Smoking has deleterious effects on the periodontal environment, including impairment of gingival ligament fibroblast function, disruption of epithelial barrier function, and attenuation of neutrophil transmigration. [2,3] (Apatzidou DA., 2022, Periodontol 2000; Yamaga et al., 2023, Sci Rep) Consequently, smoking cessation remains the most effective measure for reducing the risk of smoking-associated periodontitis.

Heated tobacco products (HTP) are relatively recently released devices that generate inhalable aerosols by heating tobacco at lower temperatures than conventional combustible cigarettes. [4] (World Health Organization, 2023) Additionally, that study noted that while some investigations have found reduced levels of many harmful substances in aerosols from HTP, reduced exposure does not correspond to a direct reduction in disease risk. Importantly, HTP aerosols may contain higher concentrations of certain constituents as compared to conventional cigarette smoke. These include propylene glycol and glycerol, used as major solvents for aerosol generation, glycidol, produced by thermal decomposition of glycerol, and butylated hydroxytoluene (BHT), an antioxidant additive. Presently, the health effects of chronic, combined exposure to these constituents remain poorly understood. [5] (Upadhyay et al., 2023, Toxics) Thus, with the rapidly increasing prevalence of HTP use, [6] (Odani S & Tabuchi T., 2023. Environ Health Prev Med) elucidation of their mechanistic effects on periodontal tissues is of critical importance. Epidemiological evidence regarding the effects of HTP on periodontal tissues remains limited, primarily due to the lack of long-term data. A large cross-sectional study conducted in Japan (JASTIS) found that HTP users had a significantly higher prevalence of self-reported periodontitis than nonsmokers. [7] (Yoshioka T & Tabuchi T., 2021, PLoS One) It is thus considered that HTP use may contribute to periodontal disease progression as an environmental modifier.

The gingival epithelium functions as a critical frontline barrier against external stimuli, such as lipopolysaccharides (LPS), which are major endotoxins of gram-negative bacteria, and chemical substances. Disruption of this epithelial barrier function contributes to the onset and progression of periodontitis. Our previous studies showed that the periodontal pathogen *Porphyromonas gingivalis* degrades the tight junction-associated proteins coxsackievirus and adenovirus receptor (CXADR) and junctional adhesion molecule 1 (JAM1), thereby allowing the pathogen to compromise the gingival epithelial barrier, resulting in increased epithelial permeability to LPS [8,9] (Takeuchi et al., 2019, PLoS Pathog; 2021 Cell Microbiol) Additionally, we have reported observations of epithelial barrier dysfunction dependent CXADR or JAM1 in gingival epithelial tissues administrated with cigarette smoke extract (CSE), neonicotinoid pesticide residues in human saliva, or in the absence of genes considered to be involved in periodontitis-related genetic disorders, such as glycogen storage disease type 1b, Kostmann syndrome, and Cohen syndrome. [3,[10], [11], [12], [13]] (Yamaga et al., 2023, Sci Rep; Tamamori et al., 2026, Toxicol Rep; Tanigaki et al., 2024, Sci Rep; Tanigaki et al., 2025, Front Cell Dev Biol; Matsumura et al., 2026, Sci Rep) These findings suggest that JAM1 and CXADR play important roles in maintaining epithelial barrier homeostasis and preventing periodontal diseases. However, the biological effects of HTP on gingival epithelial cell barrier functions have not been fully elucidated.

Assessment of histological alterations inducted by HTP in human gingival epithelial tissues is challenging, as interventional procedures such as gingival surgery and ethical considerations are required; with tissue samples usually obtained in conjunction with clinical procedures such as tooth extraction. [3] However, such opportunities are limited among younger individuals, in whom HTP use is more prevalent. [14] (Hori A, et al., 2023, Environ Health Prev Med) Accordingly, the present study investigated the effects of the three heated tobacco product aerosol extracts (HTPE), derived from IQOS, Glo, and Ploom, on gingival epithelial barrier function, with particular focus on alterations in CXADR and JAM1 expression using three-dimensional gingival tissue models.

## 2 Materials and methods

### 2.1 Cell culture

IHGE cells (epi 4; kindly provided by Shinya Murakami, The University of Osaka) were maintained in Humedia KG-2 (Kurabo), as previously described. [15] (Murakami et al., 2002, J Dental Res) Three-dimensional culturing of IHGE cells was performed as previously described, with some modifications. [8,16] (Takeuchi et al., 2019, PLoS Pathog; Nishiguchi et al., 2011, Adv Mater) Briefly, following trypsinization, IHGE cells were collected by centrifugation, then alternatively incubated for 3 min in 0.2 mg/mL^−1^ fibronectin (Sigma-Aldrich) and 0.1 mg/mL^−1^ gelatin solution (Nacalai Tesque). After three immersions, the cell surfaces were coated with fibronectin/collagen nanofilm. For tissue morphological analysis, 2 × 10^6^ cells coated with fibronectin/collagen were seeded into coverslips pre-coated with a fibronectin solution (diluted 0.2 mg/mL^−1^ in PBS) in 24-well plates (Iwaki). After 48 h of incubation, the tissues were subjected to experimental procedures and analyzed using a confocal microscope (TCS SP8; Leica Microsystems). For permeability experiments, 1 × 10^6^ cells coated with fibronectin/collagen were seeded into 24-well cell culture inserts (353096, Corning).

### 2.2 HTPE and CSE preparation

HTPE and cigarette smoke extract (CSE) were prepared as previously described. [3,17] (Gellner et al., 2016, Curr Protoc Neurosci; Yamaga et al., 2023, Sci Rep) Briefly, HTPE was prepared separately from three sticks of each commercially available HTP (IQOS, Philip Morris International; Ploom, Japan Tobacco Inc.; Glo, British American Tobacco Japan), whereas CSE was prepared from one Seven Stars cigarette (Japan Tobacco Inc.). The CSE preparation condition was based on our previous study, in which CSE prepared from one cigarette induced epithelial barrier dysfunction in IHGE cells. [3](Yamaga et al., 2023, Sci Rep) Aerosol or smoke was bubbled into 10 mL of Humedia KG-2 medium (Kurabo) using a polypropylene tube (inner diameter, 8 mm) connected to a 20-mL plastic syringe until each tobacco stick or cigarette was fully consumed, as previously reported. [3] (Yamaga et al., 2023, Sci Rep) HTPE and CSE samples were used immediately. For immunoblotting, qRT-PCR, immunocytochemistry, and epithelial barrier function assays, the existing culture medium was removed and replaced with the freshly prepared HTPE. HTPE was applied at 10 mL (10-cm culture dish), 2 mL (6-well plate), 1 mL (12-well plate), and 500 µl (12-well plate) per well, respectively.

### 2.3 LC/MS analysis

Nicotine concentrations in HTPE samples were quantified by liquid chromatography with tandem mass spectrometry (LC-MS/MS) at Infinity Lab (Yamagata, Japan; https://www.infinity-lab.jp/index.html), as previously described with modifications [10] (Tamamori et al., 2026, Toxicology Reports). Methionine sulfone was added as an internal standard. LC-MS/MS analysis was performed using an Agilent 6470B triple quadrupole mass spectrometer (Agilent Technologies, Santa Clara, CA, USA) equipped with an Agilent 1290 Infinity LC system (Agilent Technologies). Nicotine was detected in positive electrospray ionization (ESI+) mode using dynamic multiple reaction monitoring (MRM). Chromatographic separation was performed using an InfinityLab Poroshell 120 HILIC-Z column (2.1 × 100 mm, 2.7 µm, PEEK-lined; Agilent Technologies), with an injection volume of 1 µL and the column temperature maintained at 45°C. 25 mM ammonium formate in water (solvent A) and 25 mM ammonium formate in 95% acetonitrile (solvent B) were used as the mobile phase. Gradient elution was performed at a flow rate of 0.3 mL/minute as follows: 0–1.5 min, 100% solvent B; 1.5–5 min, 100–50% solvent B; and 5–6 min, 50% solvent B, followed by a post-run time of 4 min. The pH of the reagent was measured using a pH meter (F-51, HORIBA Ltd., Kyoto, Japan).

### 2.4 Antibodies, plasmids, and reagents

Antibodies, plasmids, and reagents used in this study are shown in Supplementary Table 1. Transfections were performed using FuGENE 6 Transfection Reagent (Promega), according to the manufacture’s protocol.

### 2.5 Immunoblotting and immunocytochemistry

Immunoblotting and immunocytochemistry were performed as previously described. [8,18] (Takeuchi et al., 2019, PLoS Pathog; 2021, Methods Mol Biol) Immunoreactive bands were detected using Pierce ELC Western Blotting Substrate (Thermo Scientific) and visualized with a ChemiDoc MP Imaging System (Bio Rad), then images were acquired and processed using the Image Lab Touch software package (version 2.1; Bio-Rad). Confocal microscopic images were obtained using a confocal laser microscope (TCS SP8; Leica Microsystems) equipped with a 64 × oil-immersion objective lens (numerical aperture, 1.4). Image analysis was performed using the Application Suite X software package (version 4.5.0.25531; Leica Microsystems).

### 2.6 Detection of dead cells by propidium iodide

Cell death was evaluated by propidium iodide (PI) staining, as previously described [[19], [20], [21]] (Jones and Senft, 1985; Kapuscinski, 1995; Szalai and Engedal, 2018). After HTPE treatment, unfixed cells were incubated with PI (P378, Donjindo) at a final concentration of 20 μg/mL for 15 min at room temperature in the dark. Because PI is excluded from cells with intact plasma membranes but enters membrane-compromised cells and binds to nucleic acids, PI-positive cells were considered non-viable cells. After PI staining, cells were washed with PBS and fixed with 4% paraformaldehyde in PBS (163–20145, Wako) for 10 min at room temperature. The fixed cells were treated with 0.1% TritonX-100 (168–11805, Wako) in PBS for 2 min and counterstained with DAPI (62248, Thermo Scientific) for 1 h to visualize total nuclei. Fluorescence images were acquired using confocal microscopy. Cell death was evaluated as PI-positive cells among DAPI-positive cells.

### 2.7 RNA interference

Plasmid encoding shRNA was generated by ligation of linear DNA (Sigma-Aldrich) into pSIREN-RetroQ (Clontech). The constructs pSIREN-RetroQ-shNFE2L1 #894, shNFE2L1 #1146, and shNFE2L1 #1241 were used for generation of the siRNA duplex in cells (target sequences: 5’- GTGAACACATCAGCAAGTGAAAT −3’, 5’-GCGACAGATGAAGCAGAAGGTCC-3’, and 5’-AAGCTATGTTGGATGAGATCAGC-3’, respectively). A pSIREN-RetroQ-shLuc plasmid was produced as previously described. [8,22] (Takeuchi et al., 2019, PLoS Pathog; Matsunaga et al., 2009, Nat Cell Biol) IHGE cells were transfected with shRNA-encoding plasmid using FuGENE 6 (Promega). Seventy-two hours after transfection, cells showing stable expression of shRNA were selected with puromycin (2 μg mL^−1^).

### 2.8 Quantitative real-time PCR

Reverse transcription was performed using ReverTra Ace qPCR RT Master Mix (Toyobo). Go-Taq (Promega) was used for executing PCR and quantitative real-time PCR was performed as previously described. [8] (Takeuchi et al., 2019, PLoS Pathog) Briefly, total RNA was extracted from IHGE cells using an RNeasy Micro kit (Qiagen). Complementary DNA was then synthesized with ReverTra Ace qPCR RT Master Mix (Toyobo) and real-time PCR was performed using Rotor Gene Q (Qiagen) with THUNDERBIRD SYBR qPCR Mix (Toyobo). Primer sequences are shown in Supplementary Table 2. Using the 2^−ΔΔCt^ method, the amplicon level in each sample was normalized based on the corresponding level of *β*-*ACTIN* mRNA content.

### 2.9 Prediction of transcription factor binding sites in CXADR gene promoter

Predicted transcription factor binding sites within the CXADR gene promoter were analyzed using the GeneCards database [23] (https://www.genecards.org/cgi-bin/carddisp.pl?gene=CXADR), as previously described. [12] (Tanigaki et al., 2024, Sci Rep) In addition, the TFLink gateway [24] (https://tflink.net/protein/p78310/) was used to validate the predicted associations between transcription factors and the *CXADR* promoter.

### 2.10 Epithelial barrier function assay

FITC-tracers were prepared as previously described. [8] (Takeuchi et al., 2019 PLoS Pathog) To assess the barrier functions of cell layers and tissues, *in vitro* epithelial permeability assays were performed using 12-well cell culture inserts (353180; Corning). Fluorescence intensity was determined using a VICTOR Nivo S microplate reader (PerkinElmer) and data obtained were analyzed using the Control Software package (version 5.1.0; PerkinElmer).

### 2.11 Statistical analysis

Statistical analyses were performed using the Microsoft Excel software package (Microsoft Corporation) Differences between two groups were determined using Student’s *t*-test and comparisons among multiple groups were analyzed using Dunnett’s test. A P value < 0.05 considered to indicate statistical significance.

## 3 Results

### 3.1 Three HTPE reduce CXADR in human gingival epithelial cells

To examine the effects of the three HTPs on localization of tight junction (TJ)-associated proteins, immortalized human gingival epithelial (IHGE) cells were treated with the three HTPE, then cellular localization of CXADR was analyzed using confocal microscopy. To obtain basic physicochemical information on the three HTPE preparations, nicotine concentrations and pH values were determined for the three HTPE derived from IQOS, Ploom, and Glo (Supplementary Fig. 1, Supplementary Table 3). LC/MS analysis confirmed the presence of nicotine in all three HTPE preparations, providing basic characterization data for the tobacco aerosol-derived extracts used in this study. To assess whether exposure to the three HTPE affected cell viability, cells were stained with propidium iodide (PI) and DAPI and examined by confocal microscopy. Cells treated with Triton X-100 as a positive control showed prominent PI staining, confirming effective detection of membrane-compromised cells (Supplementary Fig. 2). In contrast, at 3 h after exposure to the three HTPE PI-positive cells were negligible in both the untreated control and the three HTPE-treated groups, indicating that exposure to the three HTPE under the tested conditions did not cause substantial cell death. At this experimental condition, CXADR expression was found to be reduced by the three HTPE (Fig. 1). Additionally, confocal microscopy revealed that this CXADR reduction became progressively less pronounced with increasing dilution of the three HTPE, with weaker effects observed at 10-fold and 100-fold dilutions (Supplementary Fig. 3–5). To assess the effects of a shorter exposure period, cells were exposed to the three HTPE for 30 min. Confocal microscopy revealed a reduction in CXADR expression after the 30 min exposure, consistent with the findings observed after 3 h of exposure (Supplementary Fig. 6). To examine whether the reduction in CXADR was specific to the three HTPE exposure, we compared the effects of the HTPE with those of CSE prepared from Seven Stars, a conventional cigarette. Confocal microscopy showed that the HTPE exposure reduced CXADR expression, whereas the CSE exposure did not produce an apparent reduction in CXADR expression (Supplementary Fig. 7). To further evaluate the effect of the three HTPE on CXADR expression, reverse transcription-polymerase chain reaction (RT-PCR) and immunoblotting were performed. As shown in Figs. 2a, 2b and Supplementary Figure 8a, both *CXADR* mRNA and CXADR protein were decreased in the three HTPE-treated cells. Furthermore, western blot analysis showed that exposure to the three HTPE reduced CXADR protein expression, whereas the expression of JAM1, another tight junction-associated protein, was not markedly altered (Supplementary Fig. 9). These findings suggest that the effect of the three HTPE on tight junction-associated proteins may be relatively selective for CXADR. These findings indicate that exposure to the three HTPE selectively downregulates CXADR expression at both the transcriptional and protein levels in gingival epithelial cells.

**Fig. 1:**
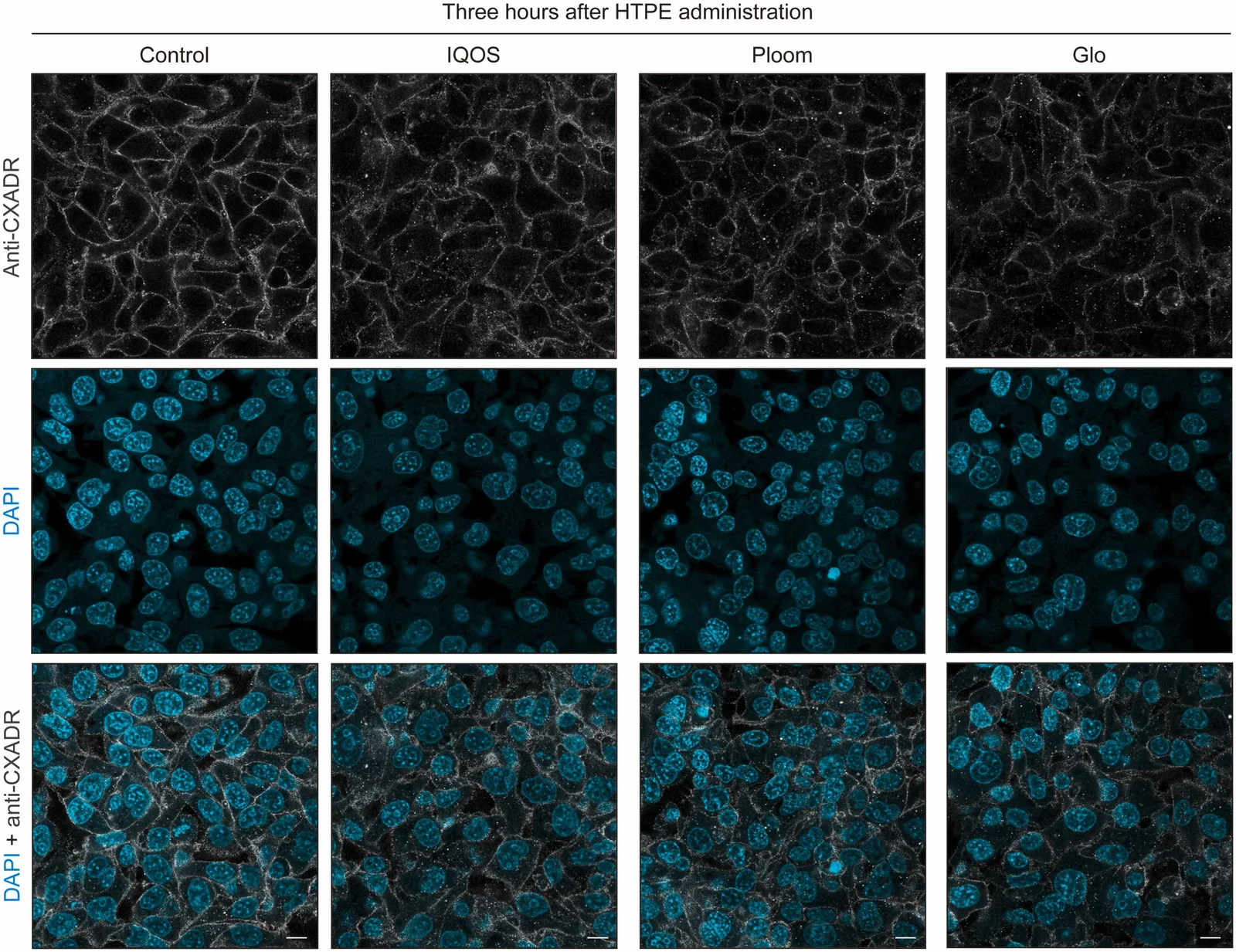
Three HTPE reduce CXADR expression in IHGE cells. IHGE cells were treated with HTPE, derived from IQOS, Ploom, or Glo, for three hours, then fixed, and stained with DAPI (cyan) and rabbit monoclonal anti-CXADR (gray: Alexa Fluor 555). Images were acquired by confocal microscopy. Scale bars, 10 μm. Data shown are representative of three independent biological replicates.

**Fig. 2:**
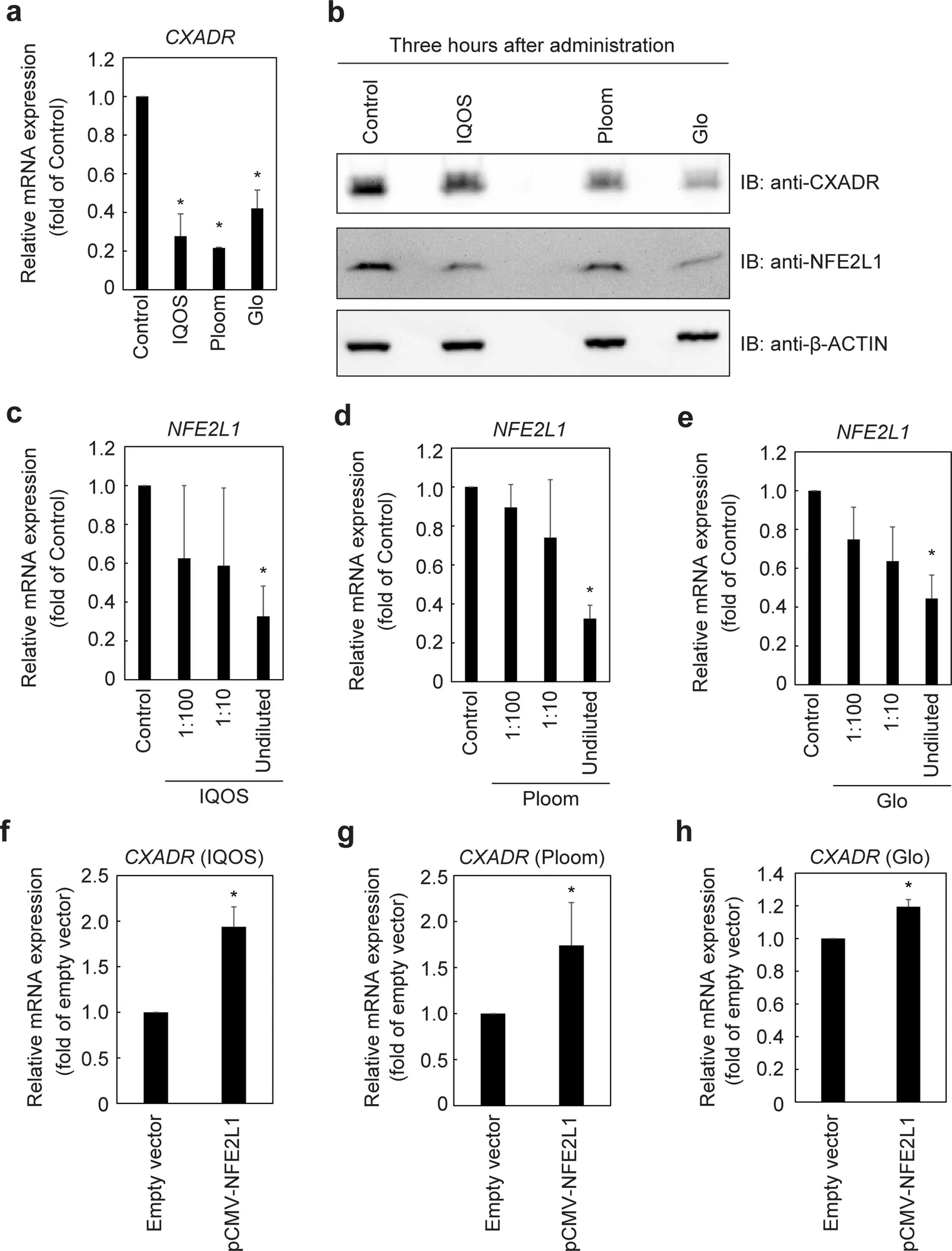
Three HTPE reduce *NFE2L1* in IHGE cells. (a) Relative *CXADR* mRNA expression in IHGE cells treated with HTPE for three hours. qRT-PCR results are expressed as fold change relative to untreated controls with three biological replicates. Statistical analyses were performed using ΔCt values by Dunnett’s test, with each heated tobacco product group compared with the control group. *P < 0.05. (b) IHGE cells were treated with HTPE for three hours and analyzed by immunoblotting with the indicated antibodies. Full-length blots are shown in Supplementary Figure 14. Data presented are representative of three biological replicates. IB, immunoblotting. (c-e) Effects of serially diluted HTPE derived from IQOS [c], Ploom [d], or Glo [e] (undiluted, 1:10 dilution, and 1:100 dilution) on *NFE2L1* mRNA expression in IHGE cells. Cells were treated with HTPE for three hours, and relative levels of *NFE2L1* mRNA expression were analyzed by qRT-PCR, expressed as fold change relative to untreated controls with three biological replicates. Statistical analyses were performed using ΔCt values by Dunnett’s test, with each heated tobacco product group compared with the control group. *P < 0.05. (f-h) Relative levels of *CXADR* mRNA expression in IHGE cells treated with the indicated HTPE derived from IQOS [f], Ploom [g], or Glo [h]. in combination with transfection of an empty or NFE2L1-coding vector. qRT-PCR results are expressed as fold change relative to the control with three biological replicates. Statistical significance was evaluated using a two-tailed Student’s *t* test. *P < 0.05.

### 3.2 Exposure to three HTPE reduces nuclear factor erythroid 2–like 1 (NFE2L1) and CXADR expression

To identify transcription factors potentially associated with the three HTPE-induced changes in CXADR expression, the GeneCards human gene database (Weizmann Institute of Science) was utilized. The analysis revealed several candidate transcription factors, such as NFE2L1, c-FOS, c-JUN, and FOSB. Immunoblotting showed that NFE2L1 was decreased by the three HTPE at three hours after administration (Fig. 2b, Supplementary Fig. 8b). To investigate the potential involvement of the transcription factor NFE2L1 in the three HTPE-induced reduction of CXADR, *NFE2L1* mRNA expression was examined by quantitative real-time PCR after exposure to undiluted, 1:10-diluted, or 1:100-diluted HTPE. *NFE2L1* expression was reduced by the three undiluted HTPE, whereas this reduction became progressively attenuated with increasing the three HTPE dilution (Figs. 2c-2e). In contrast to *NFE2L1*, *c-FOS*, *c-JUN*, and *FOSB* did not exhibit a shared reduction in expression across all three HTPE exposures (Supplementary Table 4). Among the transcription factors examined, *NFE2L1* was the only factor that showed a consistent decrease following exposure to all three HTPEs, supporting its selection for subsequent functional analyses. Based on these results, we hypothesized that NFE2L1 is also involved in the three HTPE-induced *CXADR* downregulation. To test this, IHGE cells were transfected with pCMV-NFE2L1-expressing plasmid or an empty control vector, and the expression level of *CXADR* was examined by qRT-PCR. As shown in Figs. 2f-2h, overexpression of NFE2L1 restored *CXADR* expression. These results indicate that NFE2L1 is involved in the three HTPE-induced CXADR downregulation in gingival epithelial cells.

### 3.3 Effects of NFE2L1 knockdown on CXADR expression in gingival epithelial cells

Next, the relationship between *NFE2L1* and *CXADR* expression was examined. Results of immunocytochemistry and RT-PCR analyses revealed nuclear as well as cytosolic localization of NFE2L1 in IHGE cells (Fig. 3a). To further examine this relationship, an *NFE2L1* knockdown cell line was established using an expression vector coding small hairpin RNA (shRNA) against *NFE2L1* (shNFE2L1 #894, #1146, and #1241), with firefly *luciferase* (shLuc) used as a control. Knockdown of *NFE2L1* in IHGE cells was confirmed by qRT-PCR (Fig. 3b). Under these conditions, *CXADR* expression was significantly reduced in *NFE2L1*-knockdown cells as compared with control cells (Fig. 3c), whereas the expressions of the other tight junction-related genes, including *claudin 1* (*CLDN1*), *occludin* (*OCLN*), *tight junction protein* (*TJP*) *1*, and *TJP 2*, were not markedly decreased (Supplementary Fig. 10). Next, to access the effects of *NFE2L1* knockdown on CXADR at the protein level, immunoblotting was performed and analysis conducted using confocal microscopy. Three hours after administration, reduced CXADR protein expression was confirmed (Figs. 4a, 4b, Supplementary Fig. 11). These results indicated that NFE2L1 is involved in CXADR expression in gingival epithelial cells.

**Fig. 3:**
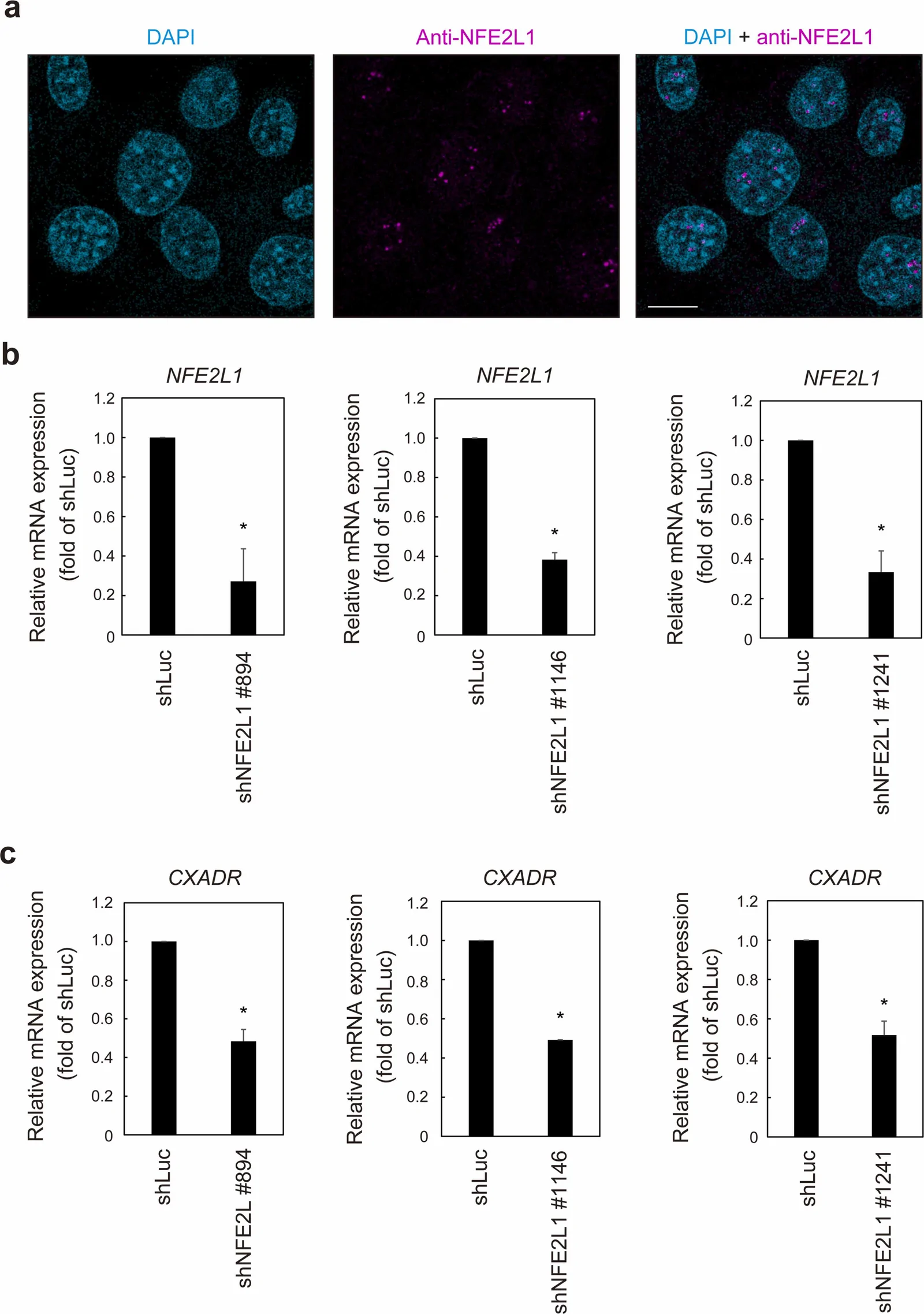
Effects of *NFE2L1* on *CXADR* expression in IHGE cells. (a) IHGE cells were fixed, stained with DAPI (cyan) and mouse monoclonal anti-NFE2L1 (magenta; Alexa Fluor 555), then analyzed using confocal microscopy. Scale bars, 10 μm. (b, c) Relative *NFE2L1* (b) and *CXADR* (c) mRNA expression levels in IHGE cells stably expressing shLuc (control), shNFE2L1 #894, shNFE2L1 #1146, or shNFE2L1 #1241. qRT-PCR results are expressed as fold change relative to shLuc with three biological replicates. Statistical significance was evaluated using a two-tailed Student’s *t* test. *P < 0.05.

**Fig. 4:**
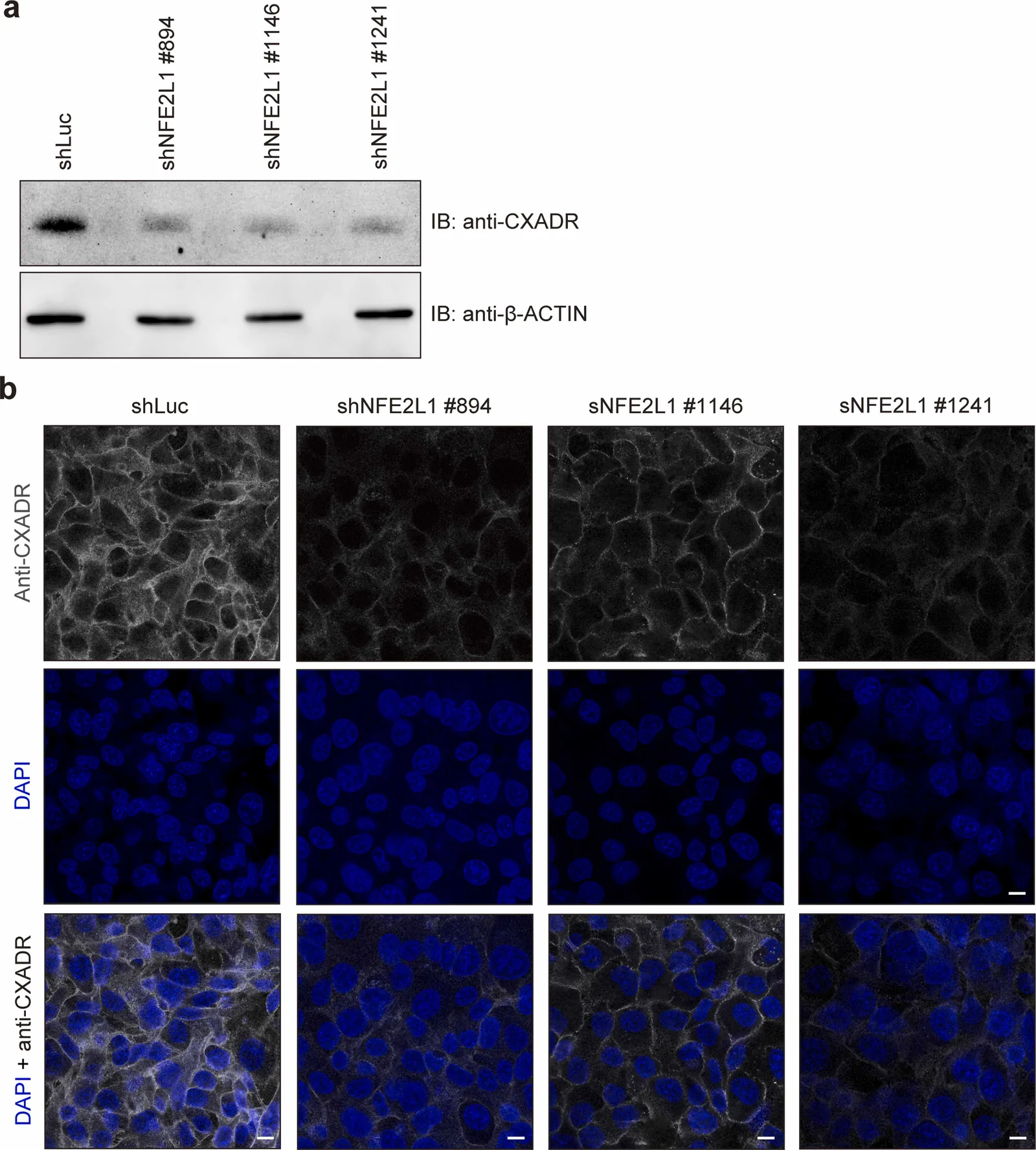
*NFE2L1* knockdown reduces CXADR in IHGE cells. (a) IHGE cells stably expressing shLuc (control), shNFE2L1 #894, shNFE2L1 #1146, or shNFE2L1 #1241 were analyzed by immunoblotting with the indicated antibodies. Full-length blots are shown in Supplementary Figure 14. Data presented are representative of three biological replicates. (b) IHGE cells stably expressing shLuc, shNFE2L1 #894, shNFE2L1 #1146, or shNFE2L1 #1241 were fixed, stained with DAPI (blue) and rabbit monoclonal anti-CXADR (gray: Alexa Fluor 555), then analyzed using confocal microscopy. Data presented are representative of three biological replicates. Scale bars, 10 μm.

### 3.4 Effects of CXADR overexpression on the three HTPE-induced impairment of gingival epithelial barrier function

To assess the effects of the three HTPE on gingival epithelial tissue barrier function, a three-dimensional tissue model was constructed using a two-layered culture system, based on a previously reported cell accumulation technique (Fig. 5a). [8,15] (Nishiguchi et al., 2011, Adv Mater; Takeuchi et al., 2019, PLoS Pathog.) Formation of gingival epithelial tissues was confirmed using confocal microscopy (Fig. 5b). Those tissues were then treated with fluorescein isothiocyanate (FITC)-labeled 40 kDa dextran or *Escherichia coli* LPS, and subjected to permeability assays. To compensate for sufficient expression of CXADR in cells treated with the three HTPE, IHGE cells overexpressing CXADR were also generated (Fig. 5b, Supplementary Fig. 12). Under these conditions, permeability assay results indicated that the three HTPE increased permeation of FITC-labeled 40 kDa dextran (Fig. 5c) and LPS (Fig. 5d), which was attenuated by CXADR overexpression. To compare the effects of the HTPE and conventional cigarette smoke on epithelial barrier function, gingival epithelial tissues were exposed to IQOS-derived HTPE or CSE prepared from Seven Stars, and permeability was assessed using 40 kDa dextran (Supplementary Fig. 13). The CSE induced a slightly greater increase in permeability than the HTPE, but negligible statistical difference compared with the control, indicating that the HTPE also increased epithelial permeability, supporting an appreciable effect of the HTPE on gingival epithelial barrier function. These findings indicate that the three HTPE impair gingival epithelial barrier function, with CXADR downregulation contributing to the increased tissue permeability to bacterial components.

**Fig. 5:**
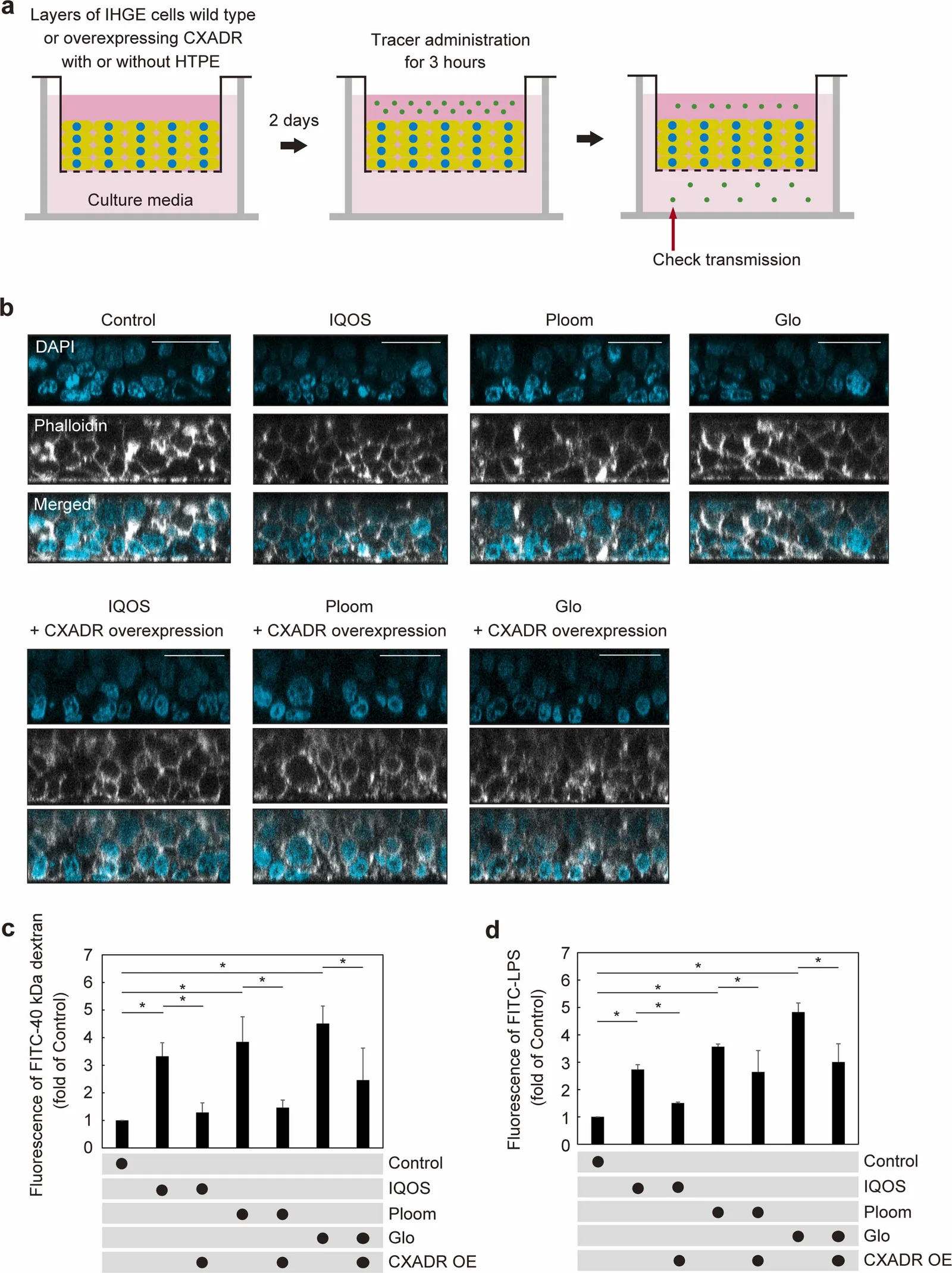
Three HTPE impair epithelial gingival epithelial barrier function in a CXADR-dependent manner. (a) Schematic image of culture-insert system. Gingival epithelial tissues with or without the three HTPE treatment were cultured in the upper compartments, then FITC-labeled tracers with or without the three HTPE were added to the culture medium. After three hours of incubation, transmission of a tracer from the upper to lower compartment was quantified using spectrometry. (b) Gingival epithelial tissue treated with or without the indicated the three HTPE and overexpression CXADR on coverslips were fixed, and stained with DAPI (cyan) and Alexa Fluor 633-conjugated phalloidin (gray), then analyzed using confocal microscopy. Scale bars, 30 μm. (c, d) Tissue permeability to FITC-dextran 40 kDa (c) or LPS (d). Results are expressed as fold change relative to the control and shown as the mean ± SD of three independent biological replicates. *P < 0.05, two-tailed Student’s *t* test (closed-testing procedure).

The results obtained indicate that the three HTPE are associated with impairment of gingival epithelial barrier function, with reduced CXADR expression potentially contributing to this effect. Reduced CXADR may contribute to increased permeability of the gingival epithelial barrier in the three HTP users.

## 4 Discussion

To the best of our knowledge, the present study is the first to investigate potential molecular mechanisms underlying the three HTP-induced impairment of gingival epithelial barrier function. Disruption of the epithelial barrier may allow translocation of environmental substances derived from HTPs into deeper tissues or blood vessels. [25,26] (Do TT et al., 2024, *Clin* Cosmet Investig Dent; Vitkov L et al., 2023, Int J Mol Sci). It is thus suggested that the three HTPs might contribute to early stages of periodontitis development. The present study should be considered pre-clinical and hypothesis-generating. Our findings identify potential molecular mechanisms by which the three HTP may affect gingival epithelial barrier function, but their clinical relevance must be interpreted in the context of existing epidemiological and clinical evidence linking the three HTP use to periodontal health. Future studies should determine whether these molecular alterations are associated with measurable outcomes in human subjects.

This study has some limitations regarding the assessment of the relationship between the physicochemical characteristics of the three HTPE and epithelial permeability. First, only three HTPs were examined. Because heated tobacco products continue to diversify, it is difficult for a single study to comprehensively represent all current and future HTPs. The primary objective of the present study was not to compare the characteristics of individual HTP products, but rather to investigate their potential effects on gingival epithelial cells. Nevertheless, the findings obtained from the three products examined here may help identify candidate molecular markers, including CXADR and NFE2L1, for future studies investigating host-cell responses to HTP-derived constituents. Second, the present in vitro model does not fully reproduce the complex oral environment, including continuous salivary flow and clearance, interactions with oral microorganisms, and other dynamic biological factors that may modify epithelial responses to the three HTP-derived constituents in vivo. In addition, the concentration of the three HTPE used in the present study cannot be directly translated to the exposure dose experienced by human gingival tissues, and the osmolality of the three HTPE preparations was not determined. Notably, IQOS-derived HTPE showed both the lowest nicotine concentration and the weakest effect on CXADR expression, suggesting that a contribution of nicotine cannot be excluded. However, the limited number of products and single nicotine measurement for each product preclude a formal assessment of this relationship.

The long-term and repeated exposure patterns associated with the three HTP use could not be fully reproduced in our in vitro gingival epithelial tissue model. In actual users, the gingival epithelium may be exposed repeatedly to heated tobacco aerosols over prolonged periods, potentially resulting in cumulative, adaptive, or reversible biological responses that differ from those observed after acute exposure. However, prolonged repeated exposure is difficult to model reliably in reconstructed gingival epithelial tissues because of frequent replacement of culture media. Nevertheless, the findings provide useful physicochemical context for interpreting the permeability data.

The present study primarily used IHGE cells, which may differ from primary gingival epithelial cells in stress responses and gene expression. On the other hand, this experimentally controlled model also provides important advantages for mechanistic studies, which enables genetic manipulation of candidate molecules, minimizes biological and environmental confounding factors, and allows loss-of-function and rescue experiments to directly examine the contribution of specific pathways to epithelial barrier function. Thus, we employed the present system to assess the molecular mechanisms underlying the three HTPE-induced impairment of gingival epithelial barrier function.

Findings obtained in the present study demonstrate that the three HTPE induce downregulation of CXADR in three-dimensional gingival epithelial tissue models. It has been shown that downregulation of CXADR expression impairs transepithelial migration of immune cells and promotes connective tissue fibrosis in lung epithelial cells. [27] (Ortiz-Zapater E et al., 2022, Nat Commun). In addition, another study found that smokers were affected by decreased numbers of leukocytes in periodontal tissues and increased fibrosis of gingival connective tissues as compared with nonsmokers. [2] (Apatzidou DA, 2022, Periodontol 2000). Taken together, the three HTPE-induced downregulation of CXADR may not only impair epithelial barrier function but also contribute to dysregulation of deeper tissue homeostasis.

Additionally, the potential involvement of NFE2L1 in CXADR expression was investigated. NFE2L1 is a transcriptional factor known to have key roles in proteasome functions, metabolic processes, and oxidative stress regulation. [28] (Zhao X et al., 2024, Life Sci). NFE2L1 downregulation in macrophages has been shown to accelerate the progression of liver fibrosis through enhanced inflammatory response, mitochondria functional impairment, and reactive oxygen species (ROS) production [29] (Lin Y et al., 2026, Theranostics). The present findings showed that treatment with the three HTPE decrease NFE2L1 expression at both the mRNA and protein levels. Although HTPs have been reported to directly generate exogenous ROS, they may also disrupt endogenous mechanisms that regulate oxidative stress. [30] (Morosini Cet al., 2025, Antioxidants).

In addition to CXADR and JAM1, gingival epithelial integrity is maintained by multiple junctional and adhesion systems. Tight junction-associated proteins such as ZO-1/TJP1 and claudins have been demonstrated in human oral epithelial tissues and contribute to the organization of the epithelial barrier [31](Imafuku et al., 2021, Cell Tissue Research). Integrins constitute another important class of epithelial adhesion molecules. In particular, α6β4 integrin interacts with laminin-5/laminin-332 and contributes to hemidesmosomal adhesion and maintenance of the dentogingival junction [32,33] (Mezawa et al., 2019, Journal of Oral Science; Mezawa et al., 2022, Cell Adhesion and Migration). Studies using human gingival epithelial cells have further shown that inflammatory stimulation can alter integrin expression or localization, indicating that this adhesion system is dynamically regulated in gingival epithelial cells. Future studies should determine whether the exposure to the three HTPE also affects integrin-mediated epithelial–matrix adhesion.

In the present study, similar trends in biological responses were observed among the three HTP examined. However, because only three products were evaluated and formal product-level comparisons were not performed, these findings cannot establish a general class effect of the three HTP. Notably, recent studies have reported that the majority of HTP users also use combusting tobacco. [34,35] (Sutanto E, et al., 2020, Int J Environ Res Public Health; Kuwabara Y, et al., 2025, BMJ Open) Furthermore, dual users are more likely to develop periodontitis than individuals who use combustible tobacco alone. [36] (Shiota C et al., 2026, Front Oral Health) Our previous study demonstrated that CSE induces translocation of JAM1, leading to increased gingival epithelial permeability. [3] (Yamaga S et al., 2023, Sci Rep) These findings raise the possibility that combined exposure may affect gingival epithelial barrier function through alterations in both CXADR and JAM1, as both are essential proteins for gingival epithelial barrier function. Future studies are required to clearly identify the impact of dual use on periodontal tissues.

## CRediT authorship contribution statement

**Shunsuke Yamaga:** Writing – original draft, Visualization, Validation, Resources, Methodology, Investigation, Funding acquisition, Formal analysis, Data curation, Conceptualization. **Keita Tanigaki:** Investigation, Formal analysis, Data curation. **Akito Sakanaka:** Resources. **Atsuo Amano:** Supervision, Funding acquisition. **Michiya Matsusaki:** Resources. **Hiroki Takeuchi:** Writing – review & editing, Visualization, Validation, Supervision, Software, Resources, Project administration, Methodology, Investigation, Funding acquisition, Formal analysis, Data curation, Conceptualization. **Masae Kuboniwa:** Supervision, Resources.

## Funding

This research was supported by a Scientific Research grant (C), number 26K12937 (to H.T.), Scientific Research grant (A), number 22H00487 (to A.A.), and Grant-in-Aid for Early-Career Scientists, numbers 24K20104 and 26K20247 (to Y. S.), from the Japan Society for the Promotion of Science. The funders had no role in study design, data collection, decision to publish, or preparation of the manuscript.

## Declaration of Competing Interest

The authors declare that they have no known competing financial interests or personal relationships that could have appeared to influence the work reported in this paper.

## Data Availability

Data will be made available on request.
