# Supplementary material for: Heated tobacco product-derived aerosol extract impairs gingival epithelial barrier function via CXADR downregulation
Source: Toxicol Rep. 2026 Sep 25;17:102346. doi: 10.1016/j.toxrep.2026.102346 (PMC13625431; doi:10.1016/j.toxrep.2026.102346)

Supplementary Table 1

Material	Source	Cat. No.
Mouse monoclonal anti-JAM1	Sigma-Aldrich	SAB4200468
Rabbit monoclonal anti-CXADR	Sino Biological	10799-R271
Mouse monoclonal anti-β-ACTIN	Sigma-Aldrich	M177-3
Mouse monoclonal anti-NFE2L1 (NRF1)	Santa Cruz	sc-28379
Alexa Fluor 555-conjugated goat anti-mouse IgG	Invitrogen	ab150114
Alexa Fluor 555-conjugated goat anti-rabbit IgG	Invitrogen	ab150078
Alexa Fluor 633 phalloidin	Thermo Fisher Scientific	A22284
Goat anti-mouse antibody conjugated to horseradish peroxidase	Cell Signaling Technology	7076
Goat anti-rabbit antibody conjugated to horseradish peroxidase	Cell Signaling Technology	7074
pCMV-HA-inserted CXADR	Takeuchi *et al* ., 2021	-
pIRES-Puro-HA-inserted CXADR	Takeuchi *et al* ., 2021	-

Supplementary Table 2

Primers	Sequences (5' to 3')	Anealing temperature, time (cycles)
β-ACTIN forward	GCATGGGTCAGAAGGATTCCT	58℃, 15 seconds (45)
β-ACTIN reverse	TCGTCCCAGTTGGTGACGAT	58℃, 15 seconds (45)
CXADR forward	CAGTGCCTGTTGCGTCTAAA	58℃, 15 seconds (45)
CXADR reverse	CTTTGGAGGTGGCACATCTT	58℃, 15 seconds (45)
NFE2L1 forward	GGAACAGCAGTGGCAAGATCTC	58℃, 15 seconds (45)
NFE2L1 reverse	GCAAGGCTGTAGTTGGTGCTCA	58℃, 15 seconds (45)
c-FOS forward	AGAATCCGAAGGGAAAGGAA	58℃, 15 seconds (45)
c-FOS reverse	CTTCTCCTTCAGCAGGTTGG	58℃, 15 seconds (45)
c-JUN forward	CCCCAAGATCCTGAAACAGA	58℃, 15 seconds (45)
c-JUN reverse	ATAATCCAGTCCAGCAACGG	58℃, 15 seconds (45)
FOSB forward	AGGAAGAGGAGAAGCGAAGG	58℃, 15 seconds (45)
FOSB reverse	CTTCGTAGGGGATCTTGCAG	58℃, 15 seconds (45)
CLDN1 forward	CCGTTGGCATGAAGTGTATG	58℃, 15 seconds (45)
CLDN1 reverse	CCAGTGAAGAGAGCCTGACC	58℃, 15 seconds (45)
OCLN forward	ATGGCAAAGTGAATGACAAGCGG	58℃, 15 seconds (45)
OCLN reverse	CTGTAACGAGGCTGCCTGAAGT	58℃, 15 seconds (45)
TJP1 forward	CTCAAGAGGAAGCTGTGGGTA	58℃, 15 seconds (45)
TJP1 reverse	AGGGTTTTCCTTGGCTGACA	58℃, 15 seconds (45)
TJP2 forward	ATTAGTGCGGGAGGATGCCGTT	58℃, 15 seconds (45)
TJP2 reverse	TCTGCCACAAGCCAGGATGTCT	58℃, 15 seconds (45)

Supplementary Table 3

HTPEs	Nicotine concentration (μM)	pH
IQOS (3 sticks)	476	7.59 ± 0.03
Ploom (3 sticks)	1,443	7.66 ± 0.01
Glo (3 sticks)	1,519	7.59 ± 0.01
Humedia KG2 (Control)	Not Detected	7.50 ± 0.04

Supplementary Table 4

Transcription factor	Control	IQOS (mean ± SD)	Ploom (mean ± SD)	Glo (mean ± SD)
NFE2L1	1	0.326 ± 0.156*	0.324 ± 0.069*	0.444 ± 0.121*
c-FOS	1	0.465 ± 0.085*	0.681 ± 0.044	1.272 ± 0.371
c-JUN	1	1.305 ± 0.075	3.161 ± 0.329*	5.291 ± 0.725*
FOSB	1	1.267 ± 0.387	2.474 ± 1.080*	4.660 ± 2.060*

Supplementary Figure 1

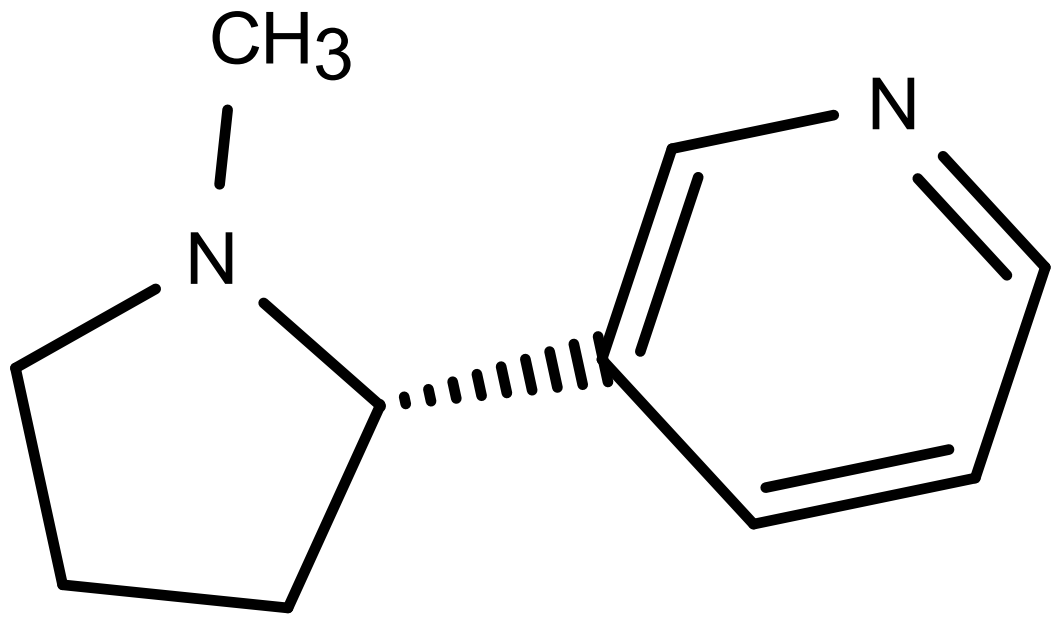

Supplementary Figure 2

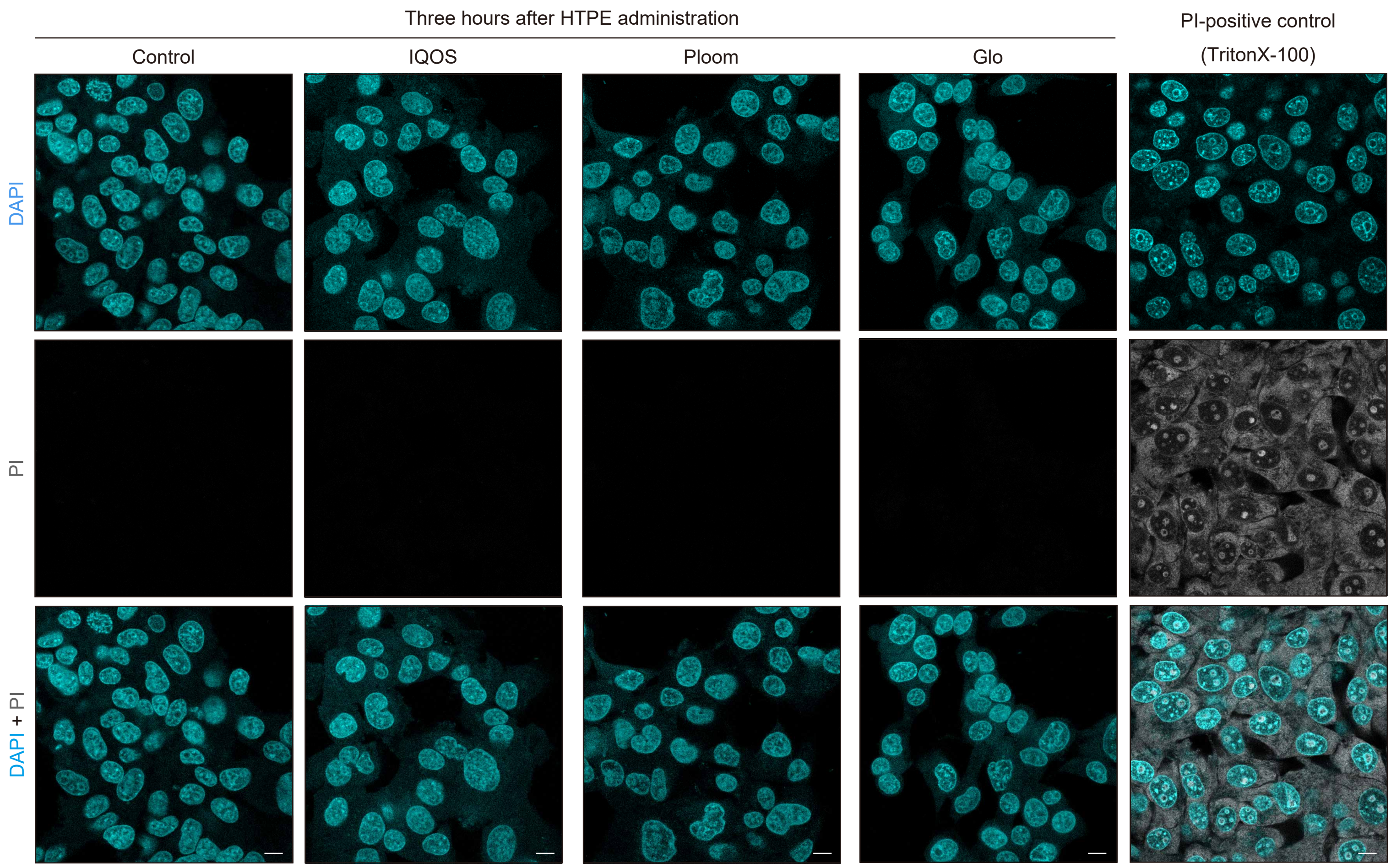

Supplementary Figure 3

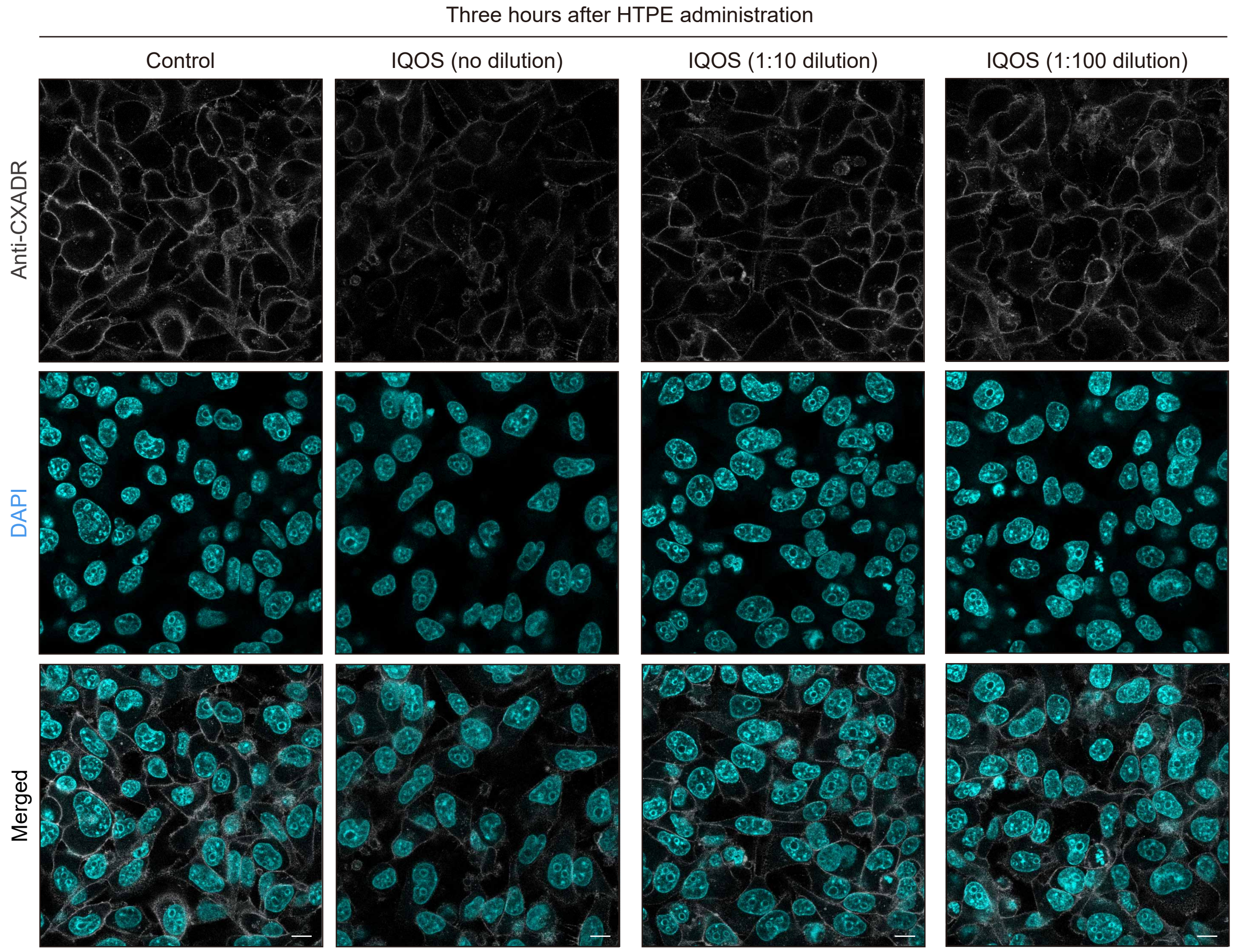

Supplementary Figure 4

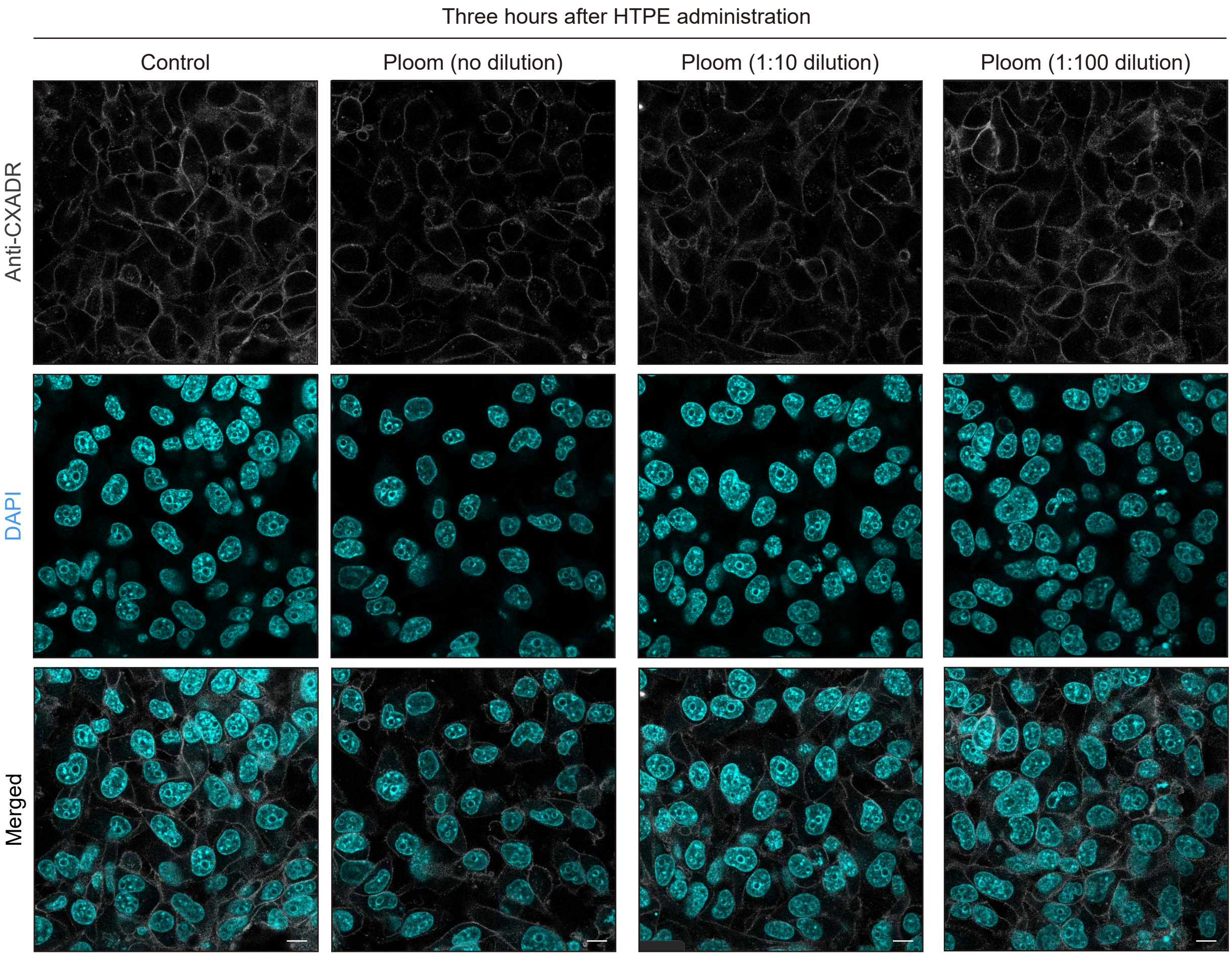

Supplementary Figure 5

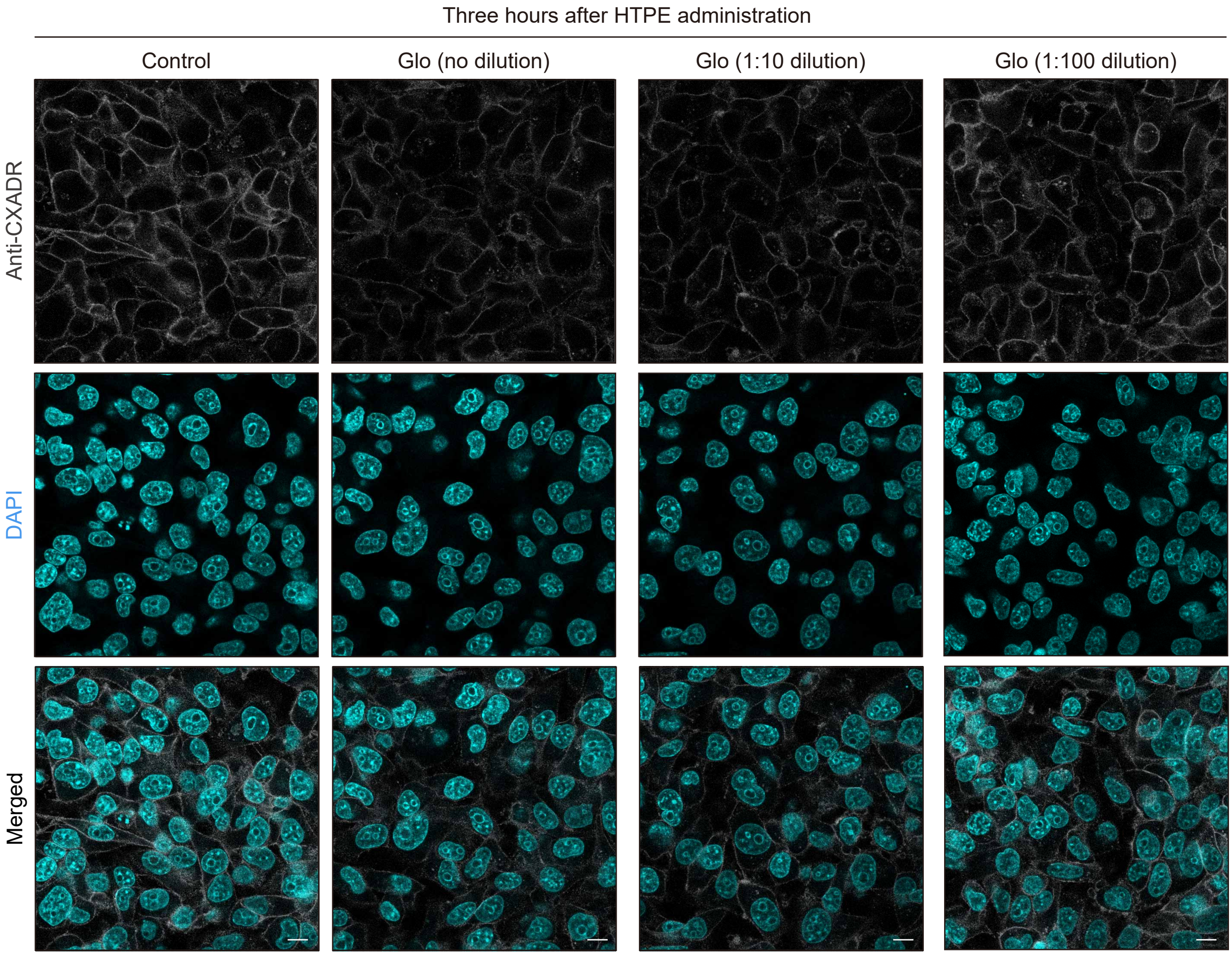

Supplelentary Figure 6

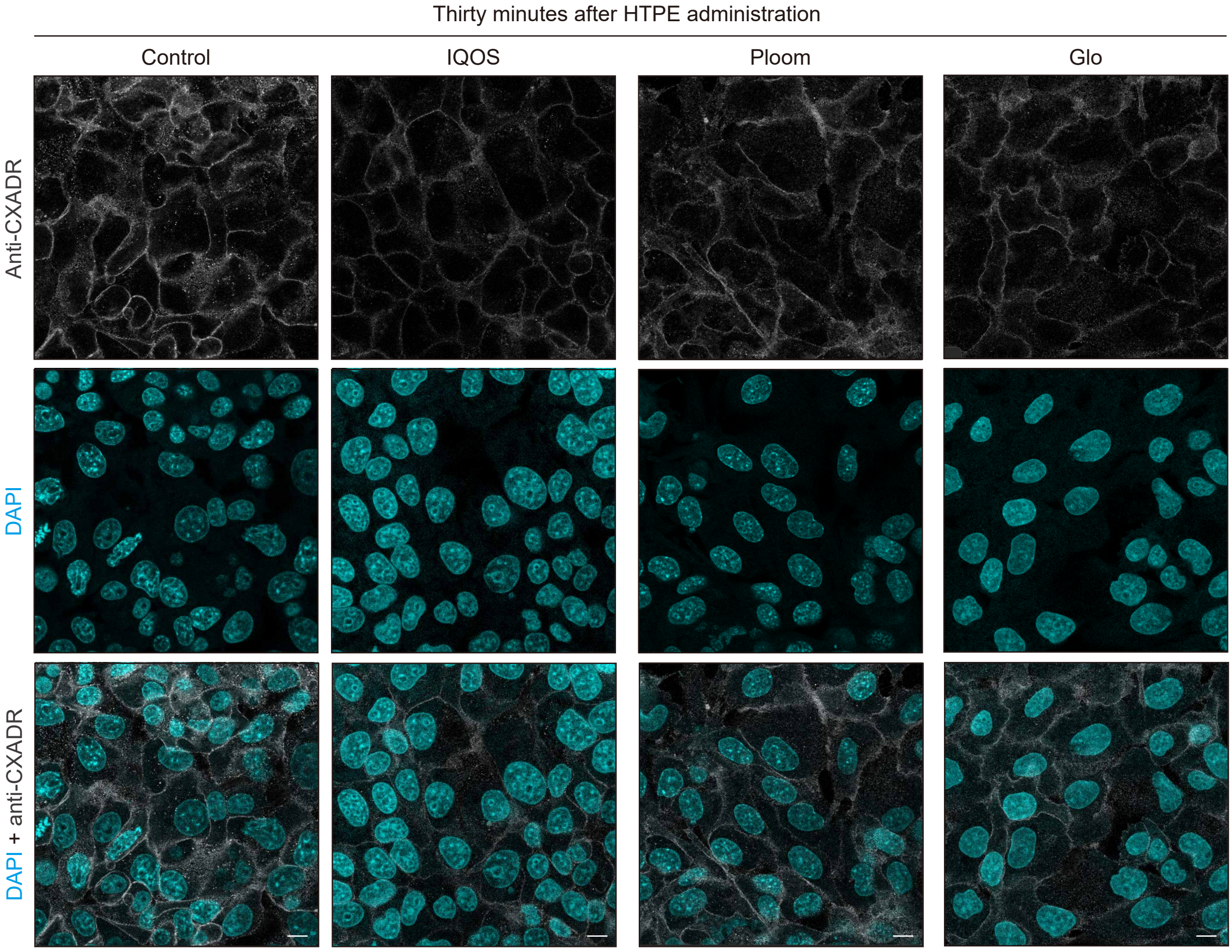

Supplementary Figure 7

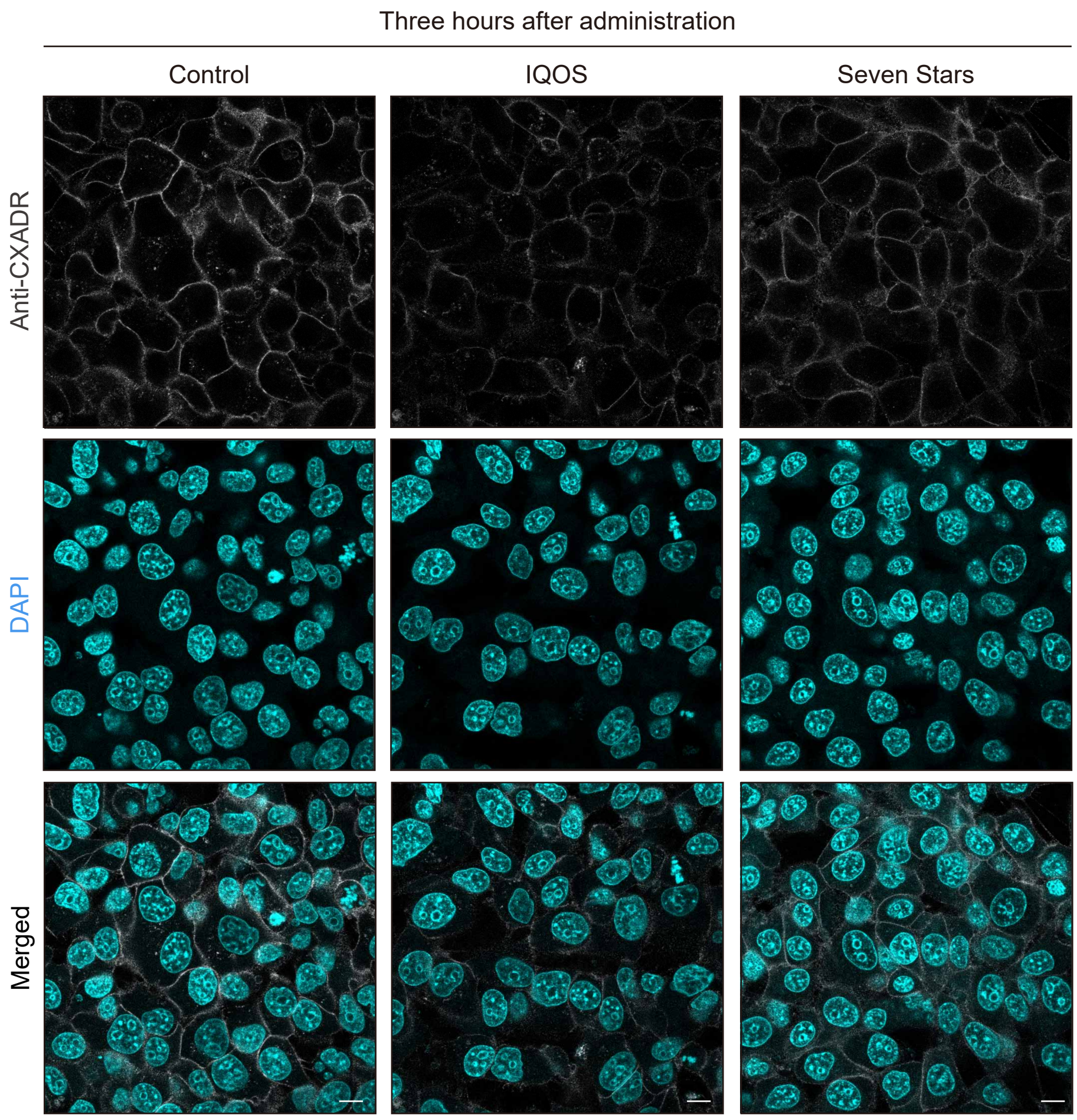

Supplementary Figure 8

a

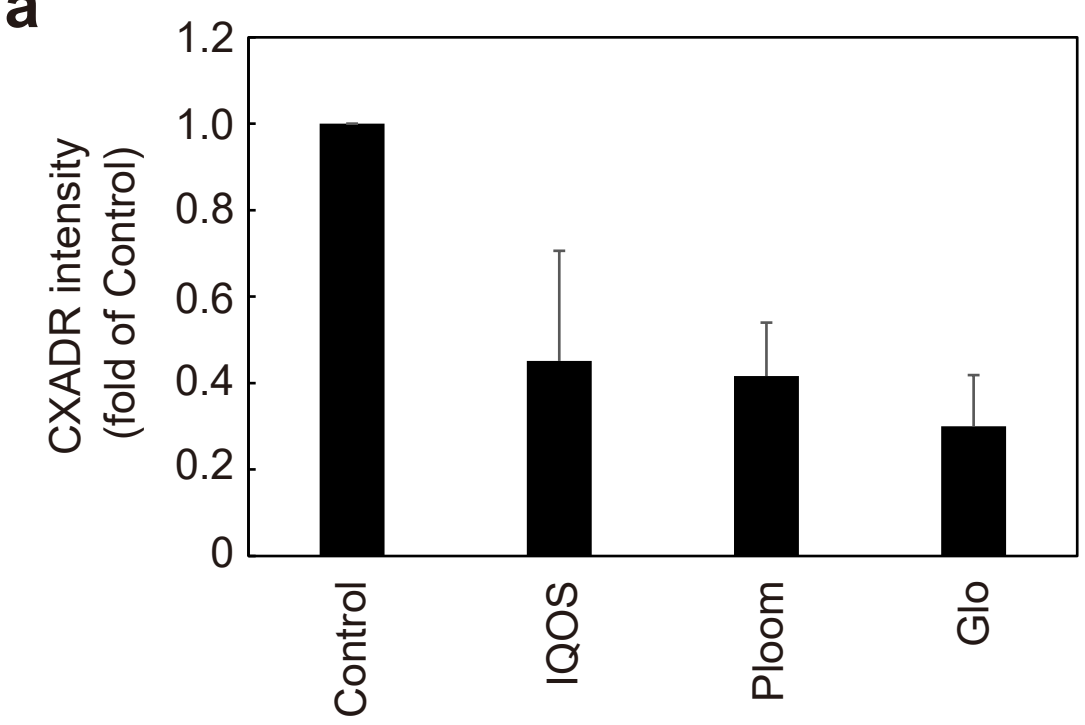

b

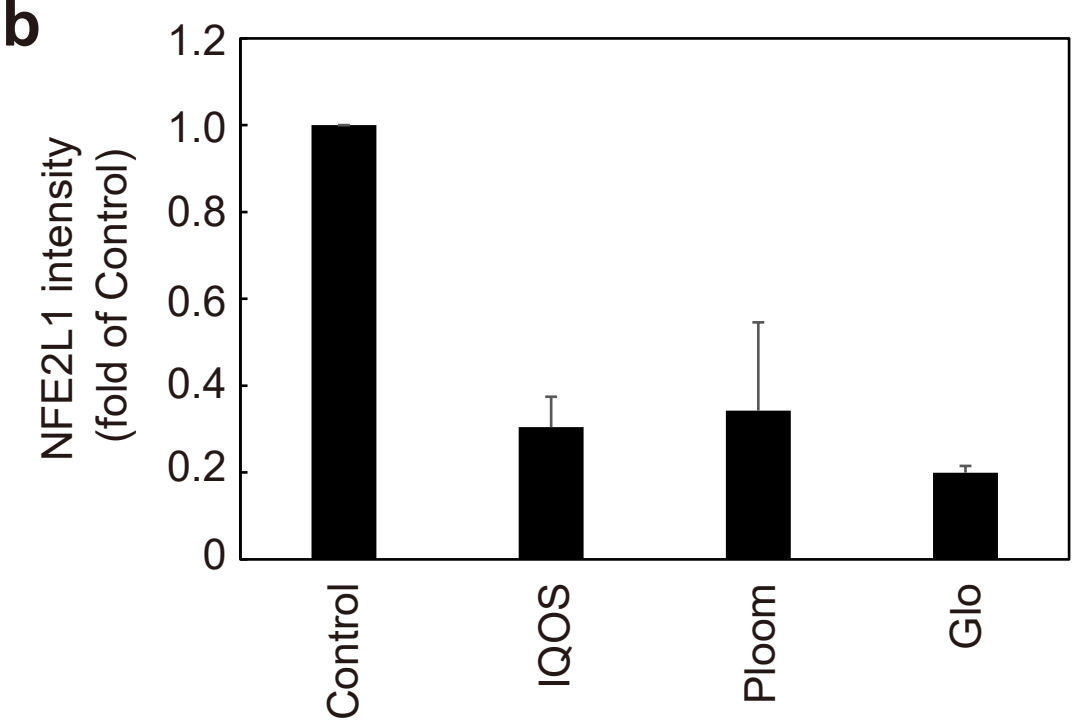

Supplementary Figure 9

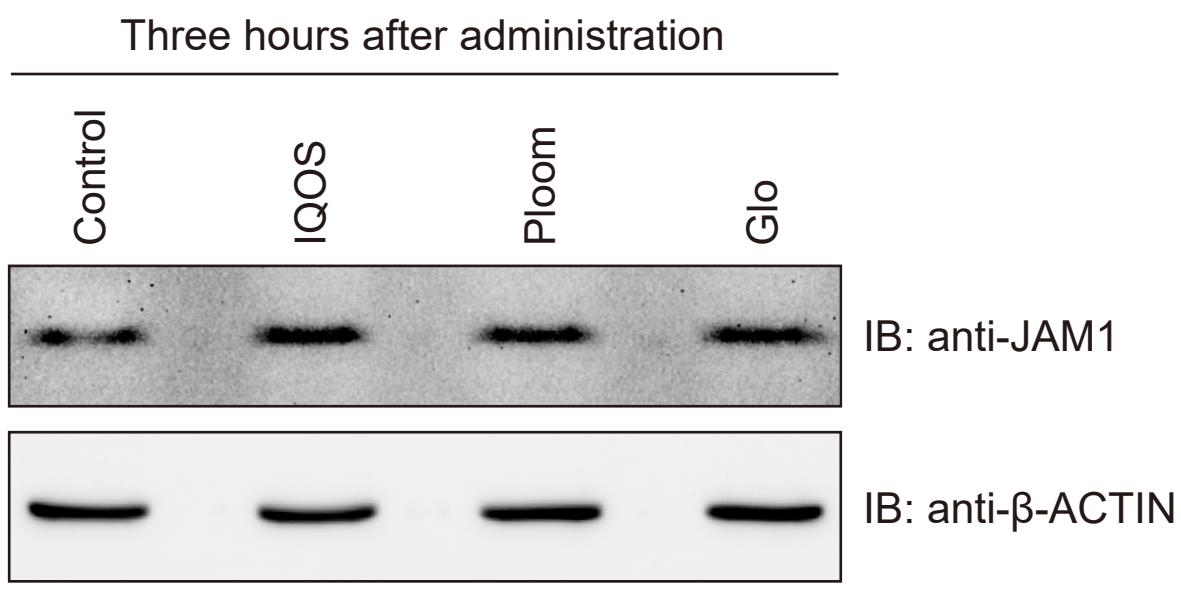

Supplementary Figure 10

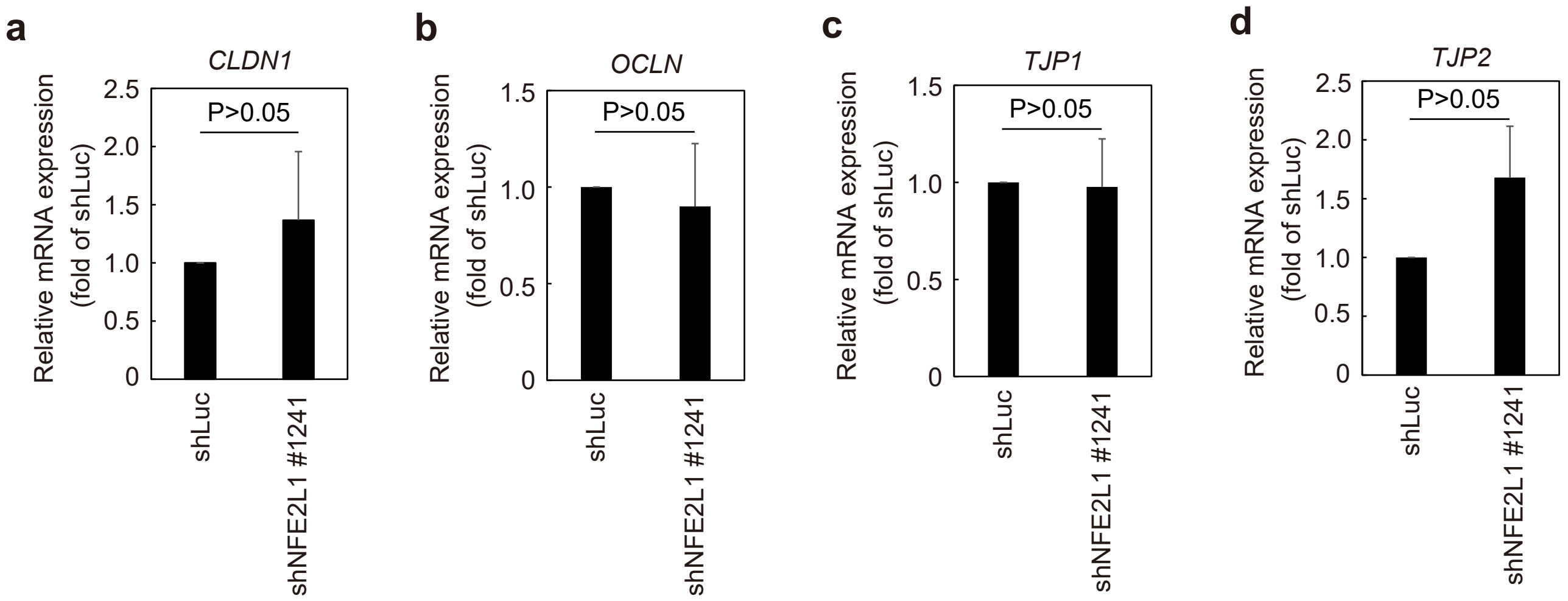

Supplementary Figure 11

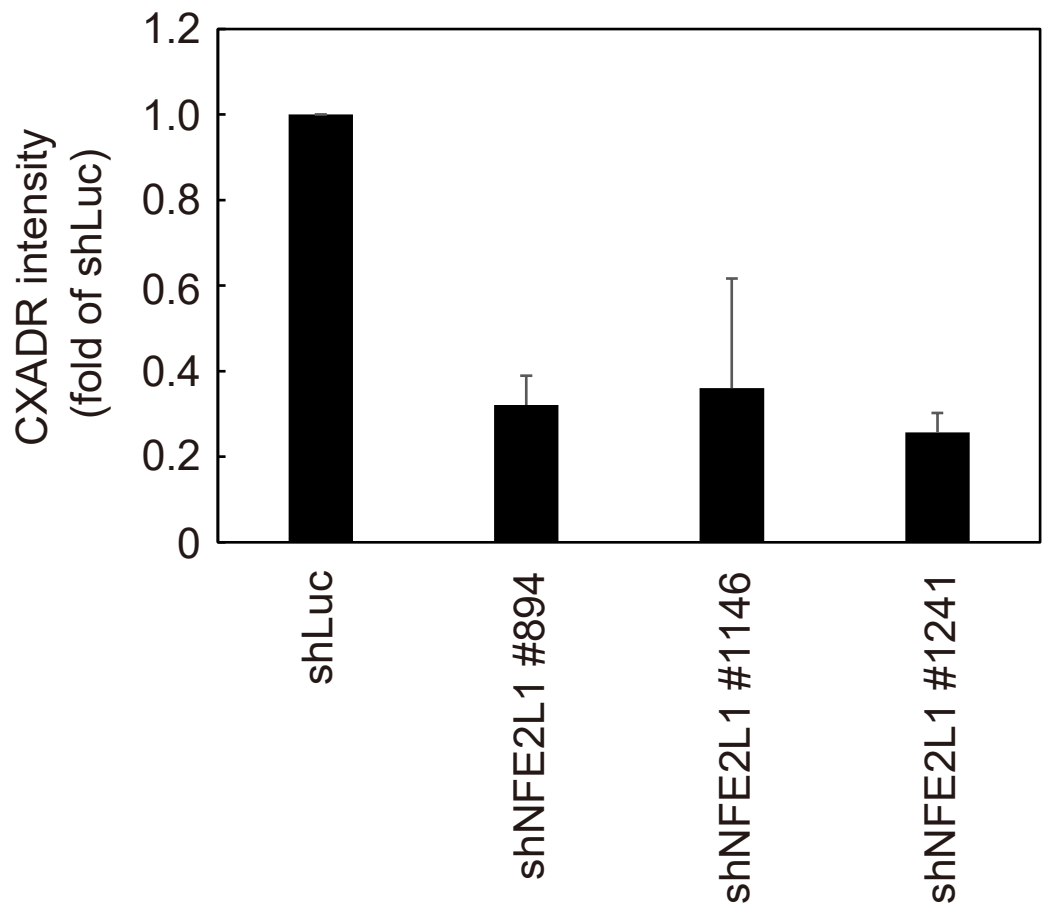

Supplementary Figure 12

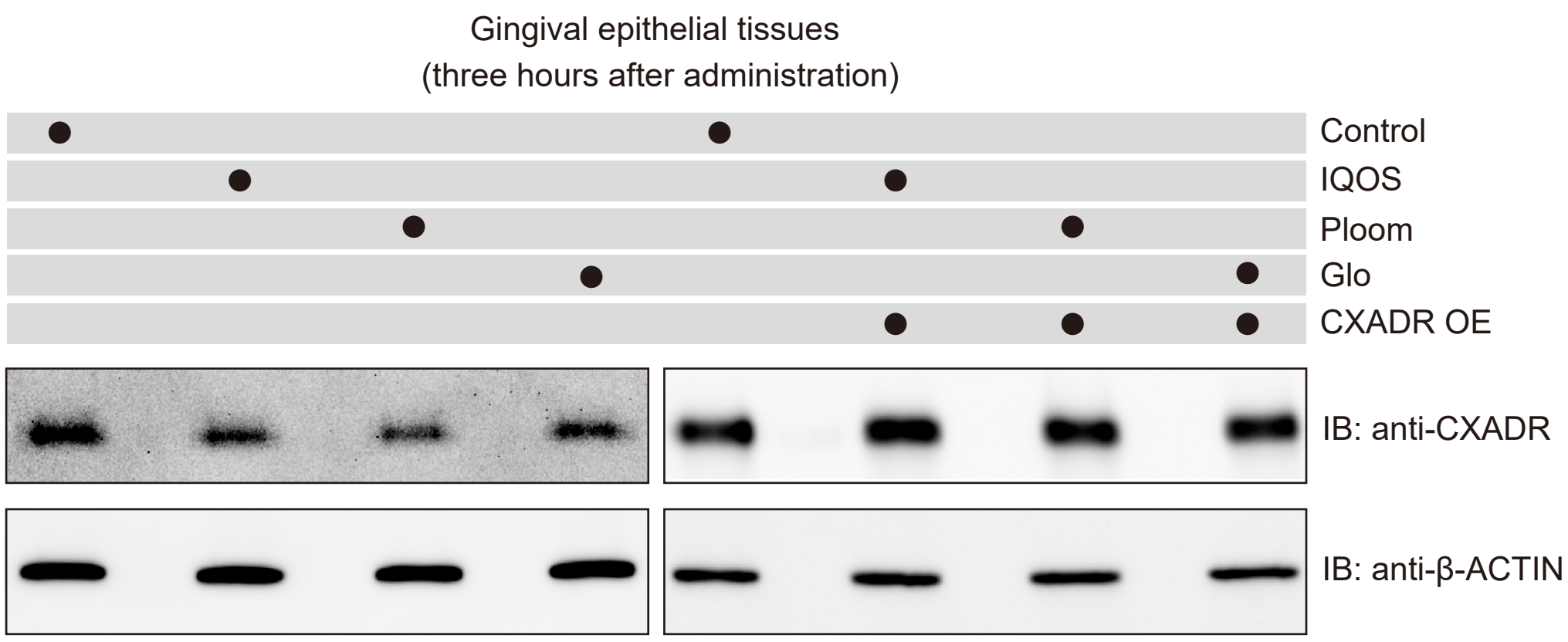

Supplementary Figure 13

a

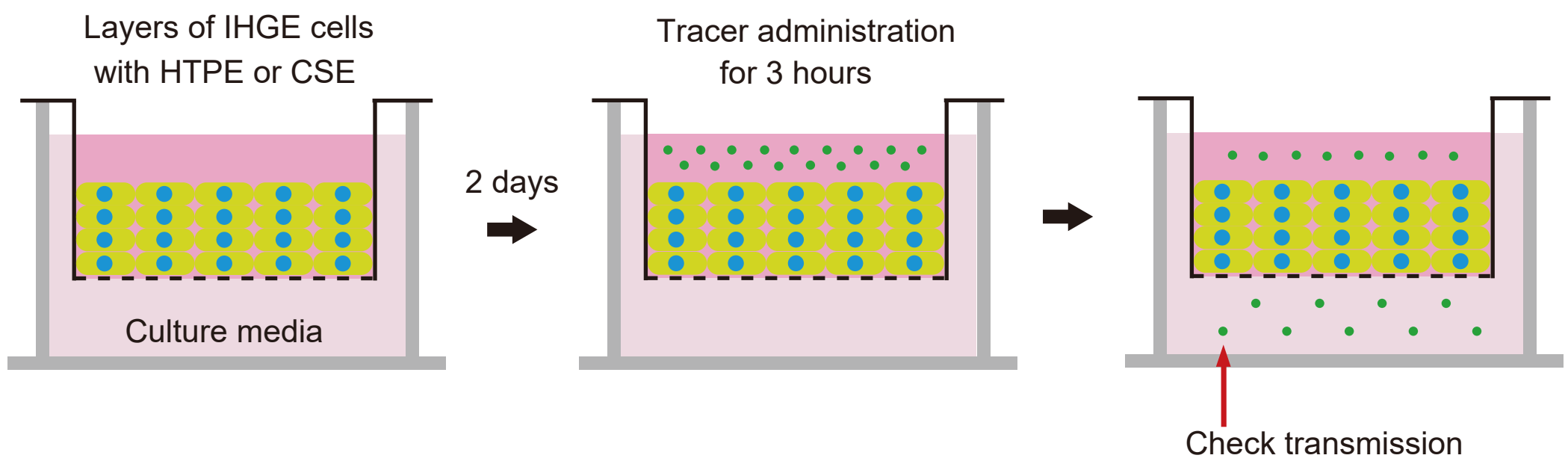

b

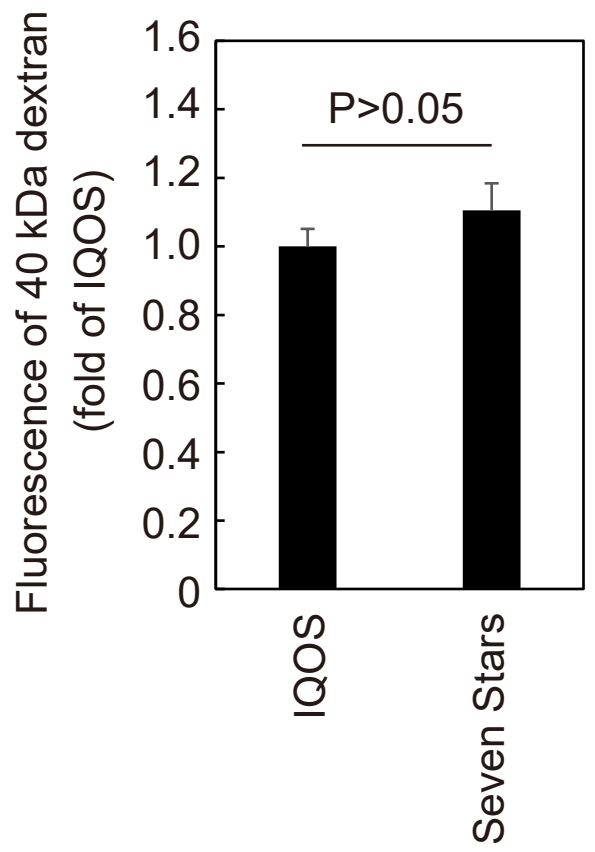

Supplementary FIgure 14. Immunoblots performed in this study.

Figure 2b

IB anti-CXADR

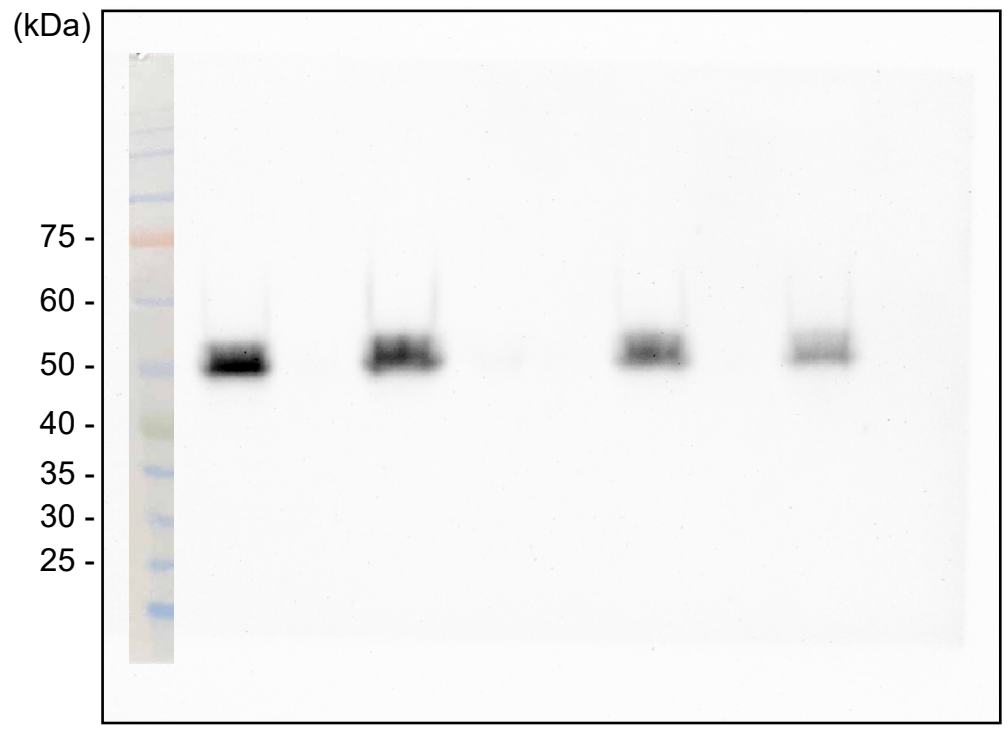

Membane

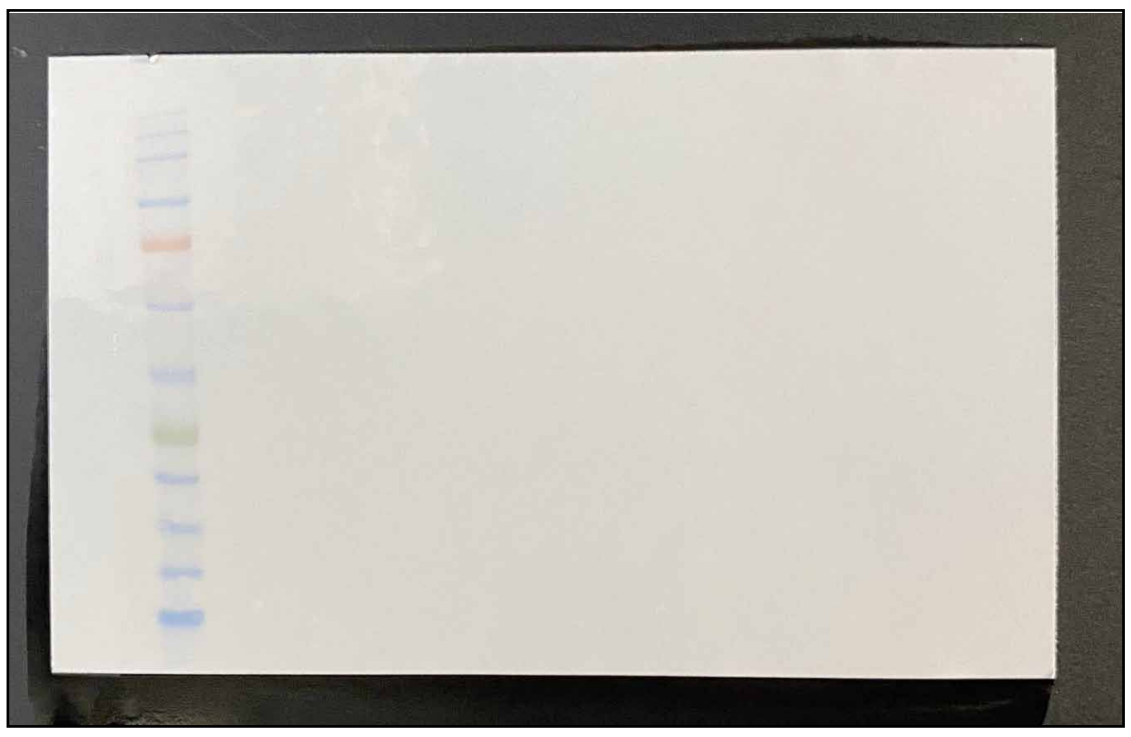

Chemiluminescence (uncropped)

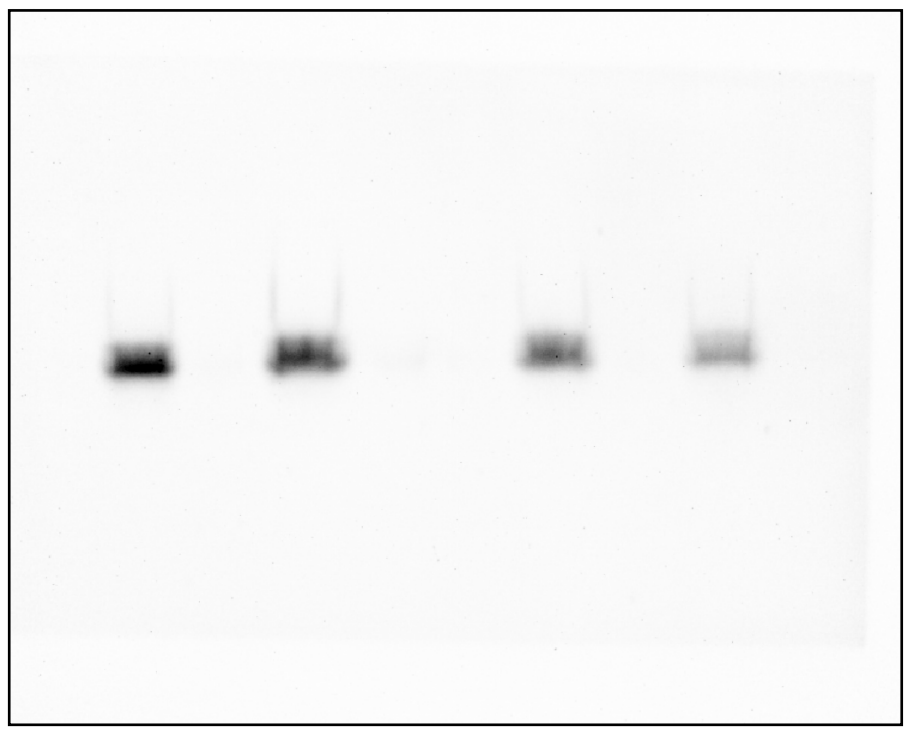

IB anti-NFE2L1

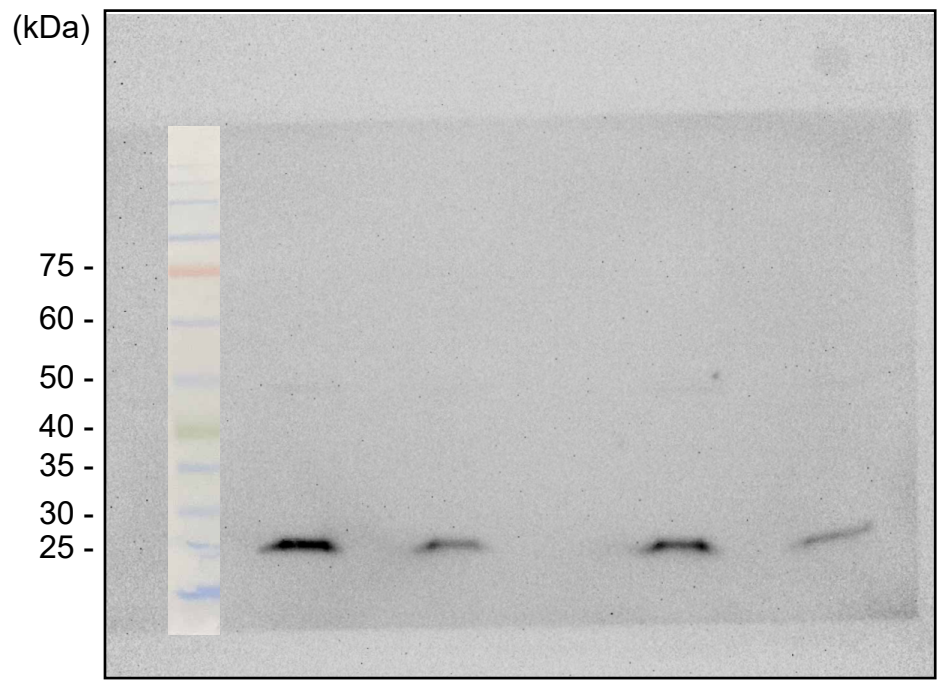

Membane

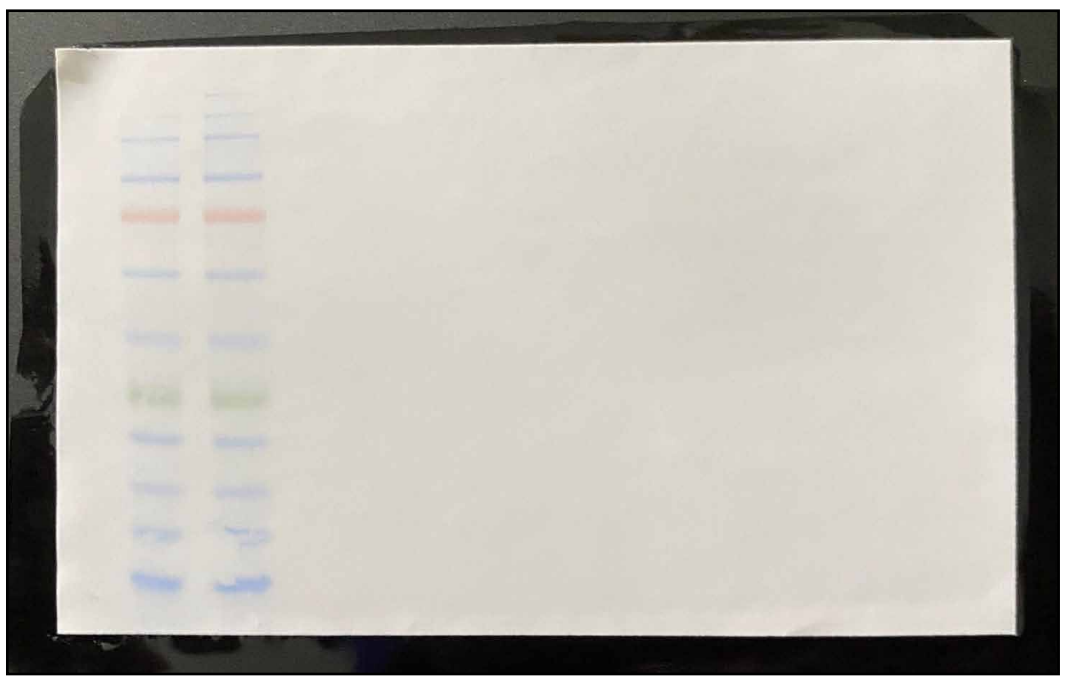

Chemiluminescence (uncropped)

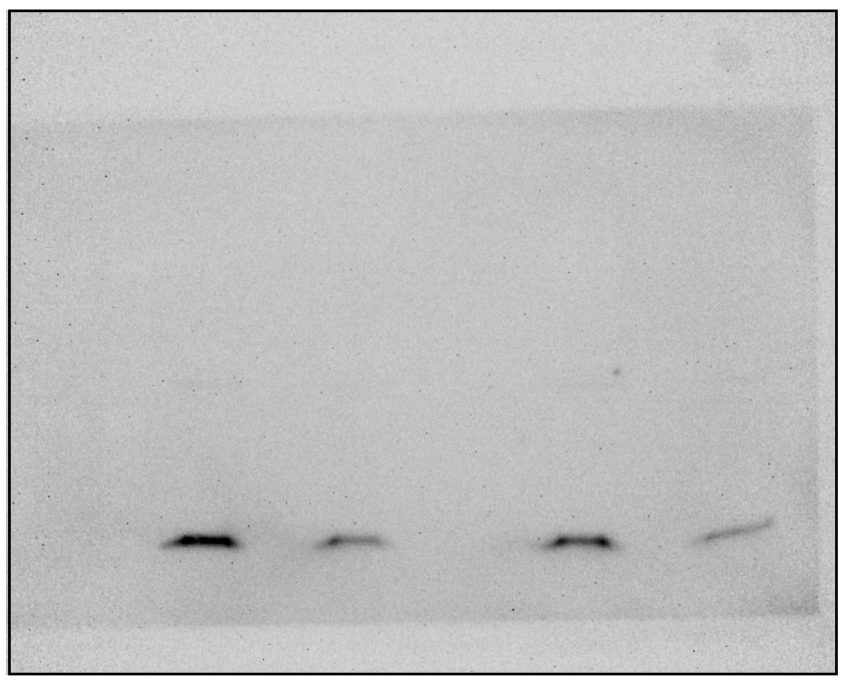

IB anti-β-ACTIN

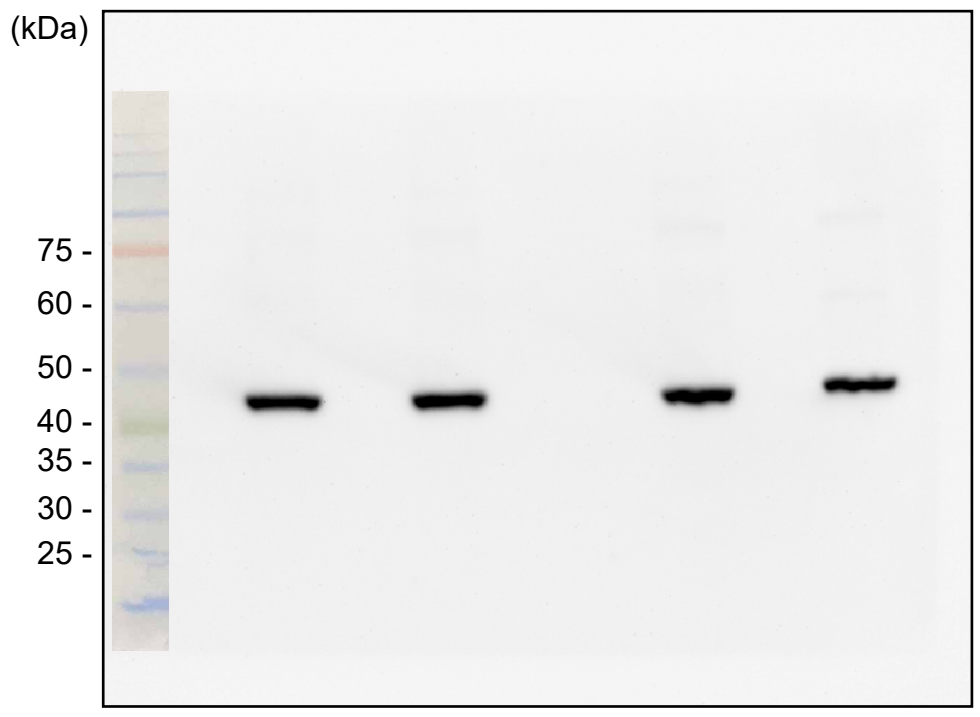

Membane (same as the anti-NFE2L1 membrane)

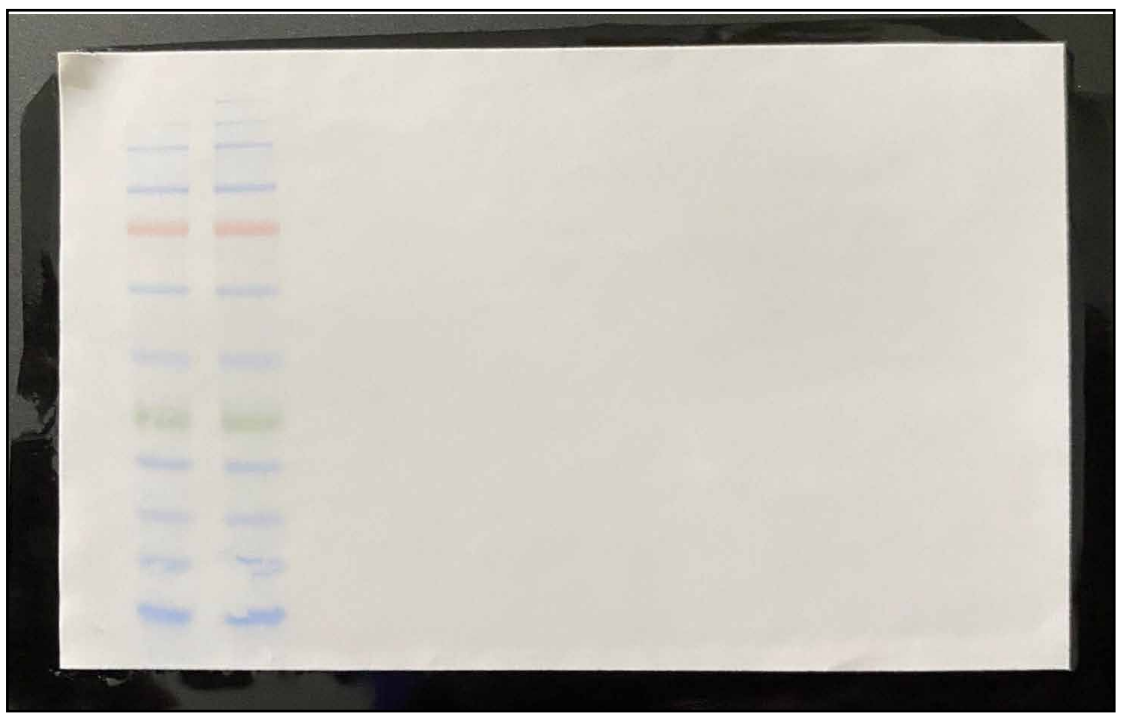

Chemiluminescence (uncropped)

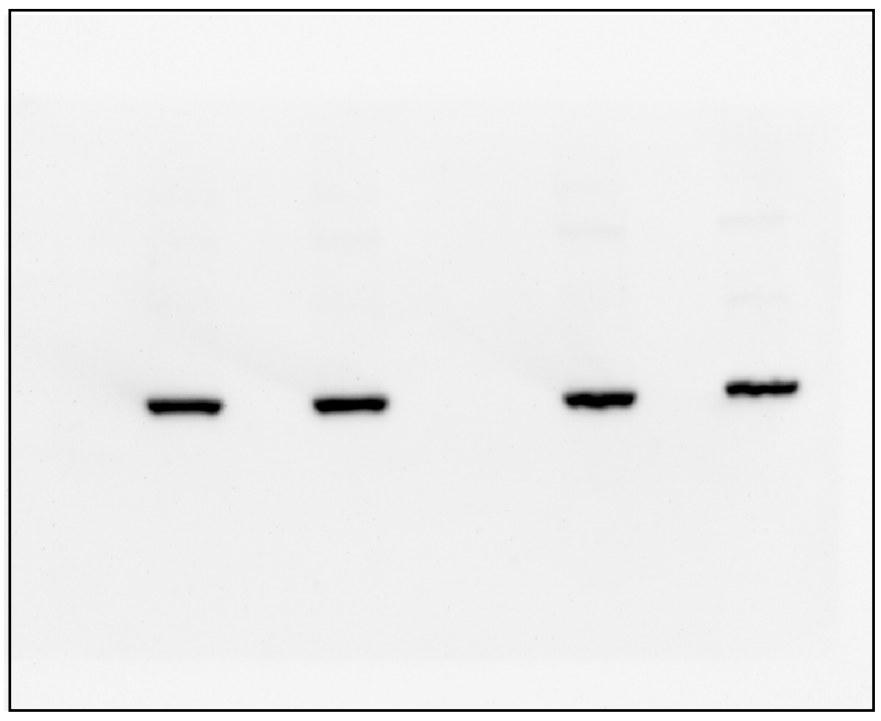

Figure 4a

IB: anti-CXADR

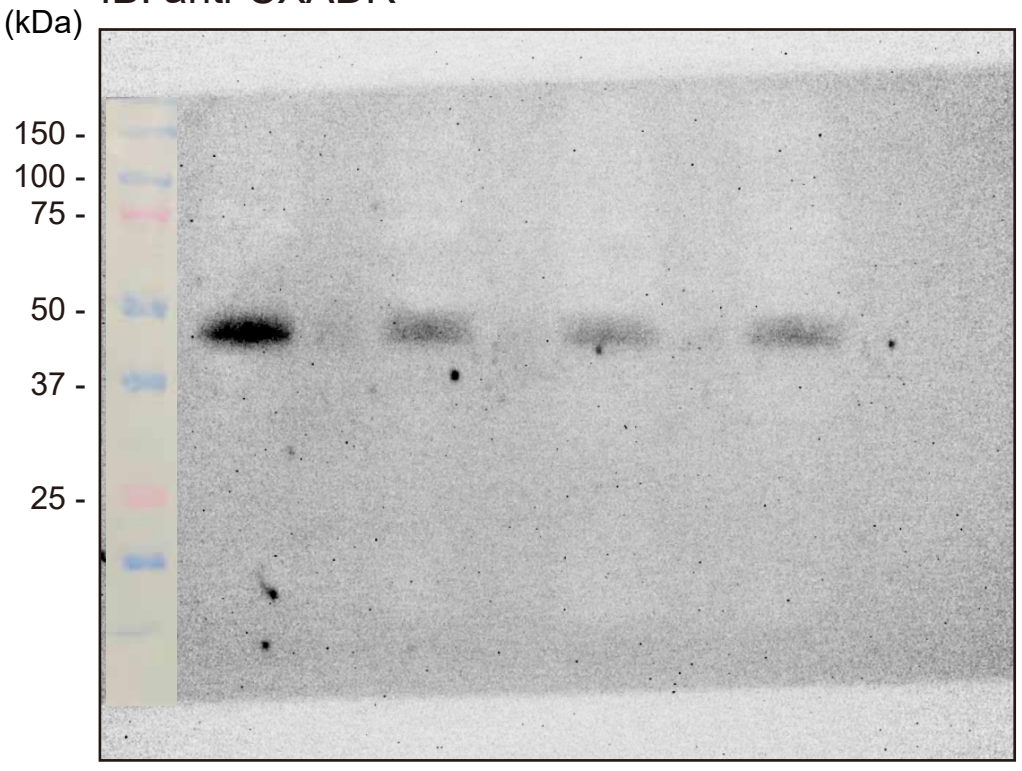

Membrane

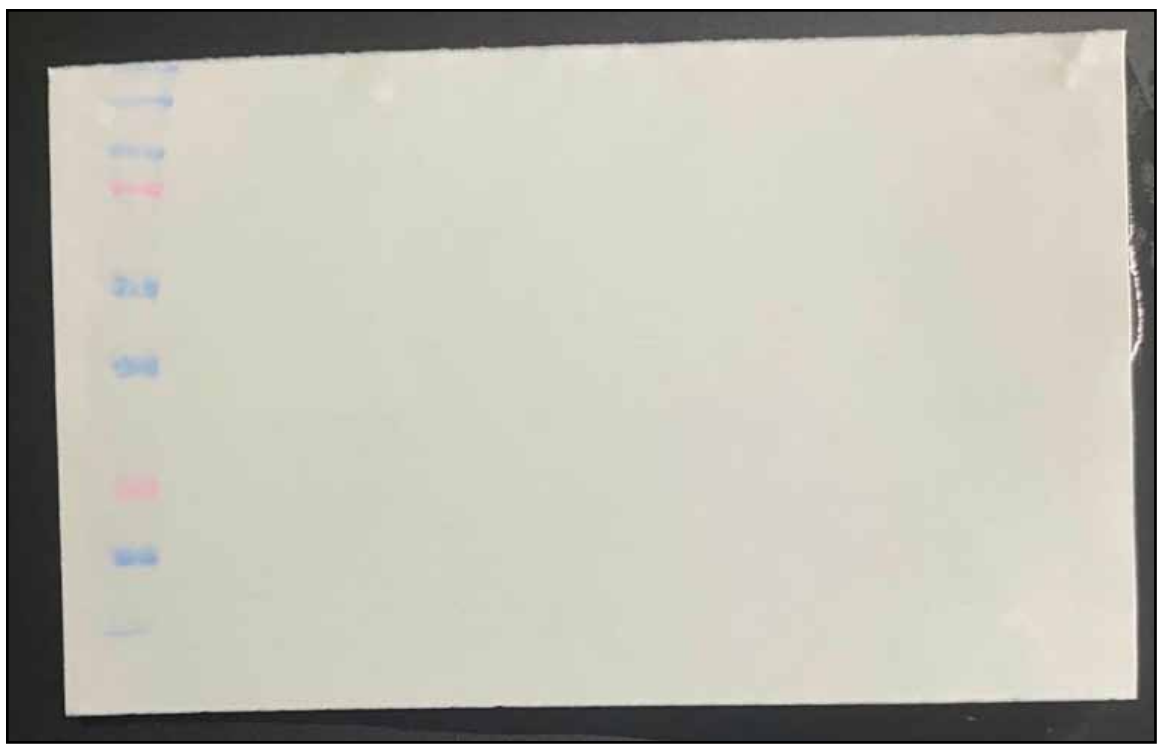

Chemiluminescence (uncropped)

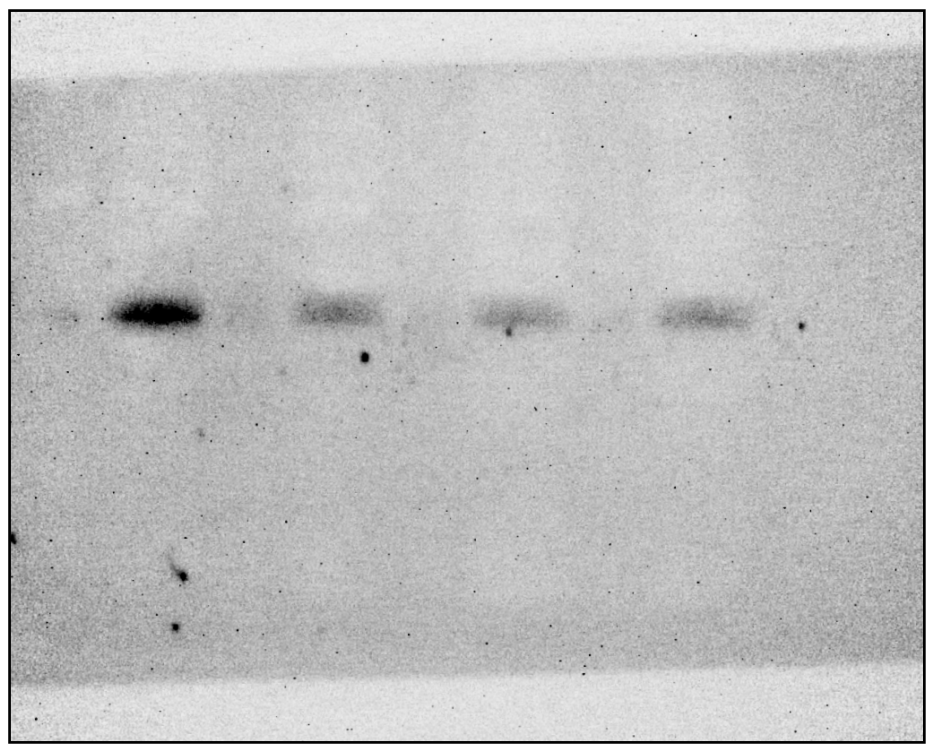

IB: anti-β-ACTIN

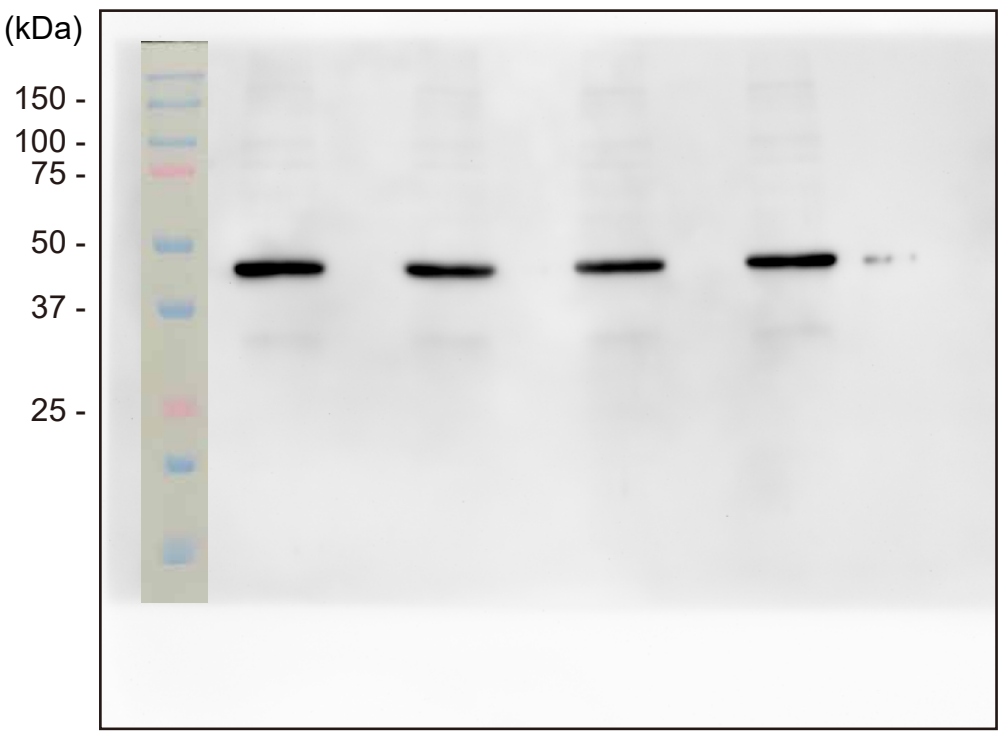

Membrane

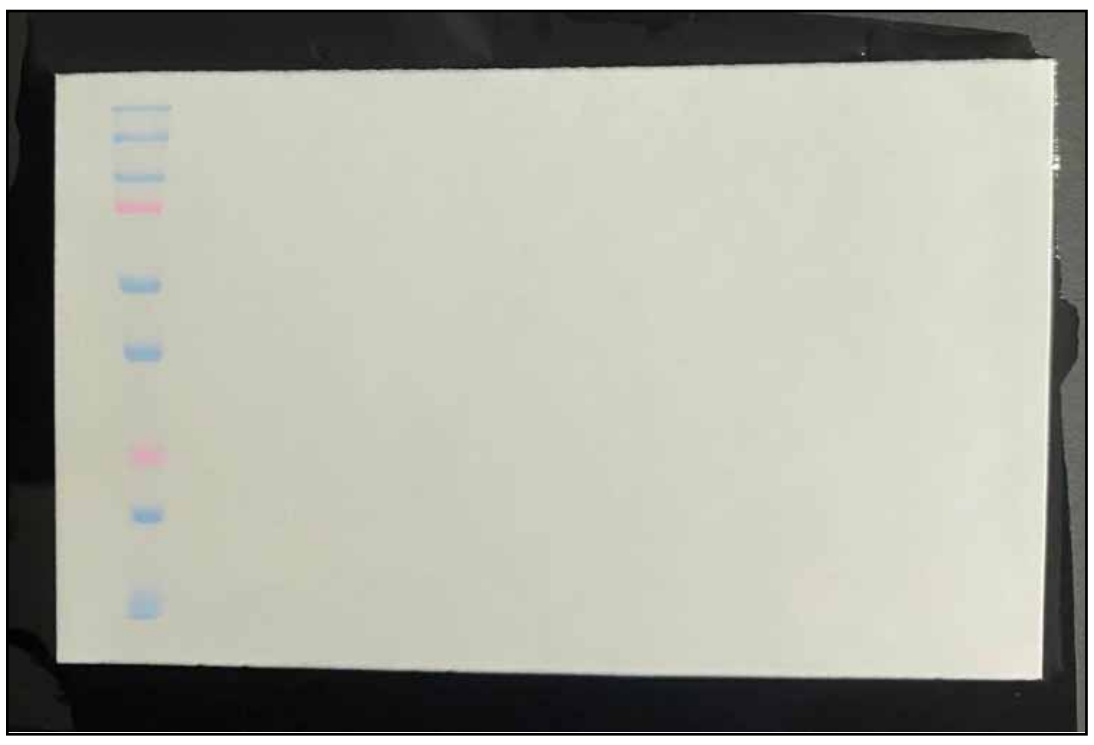

Chemiluminescence (uncropped)

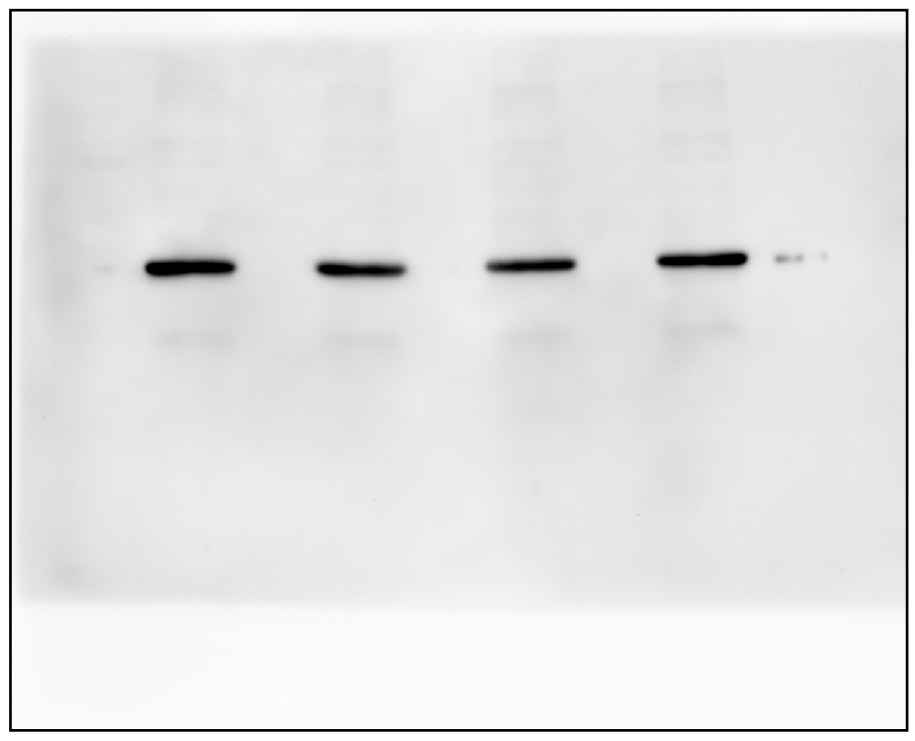

Supplementary Figure 9

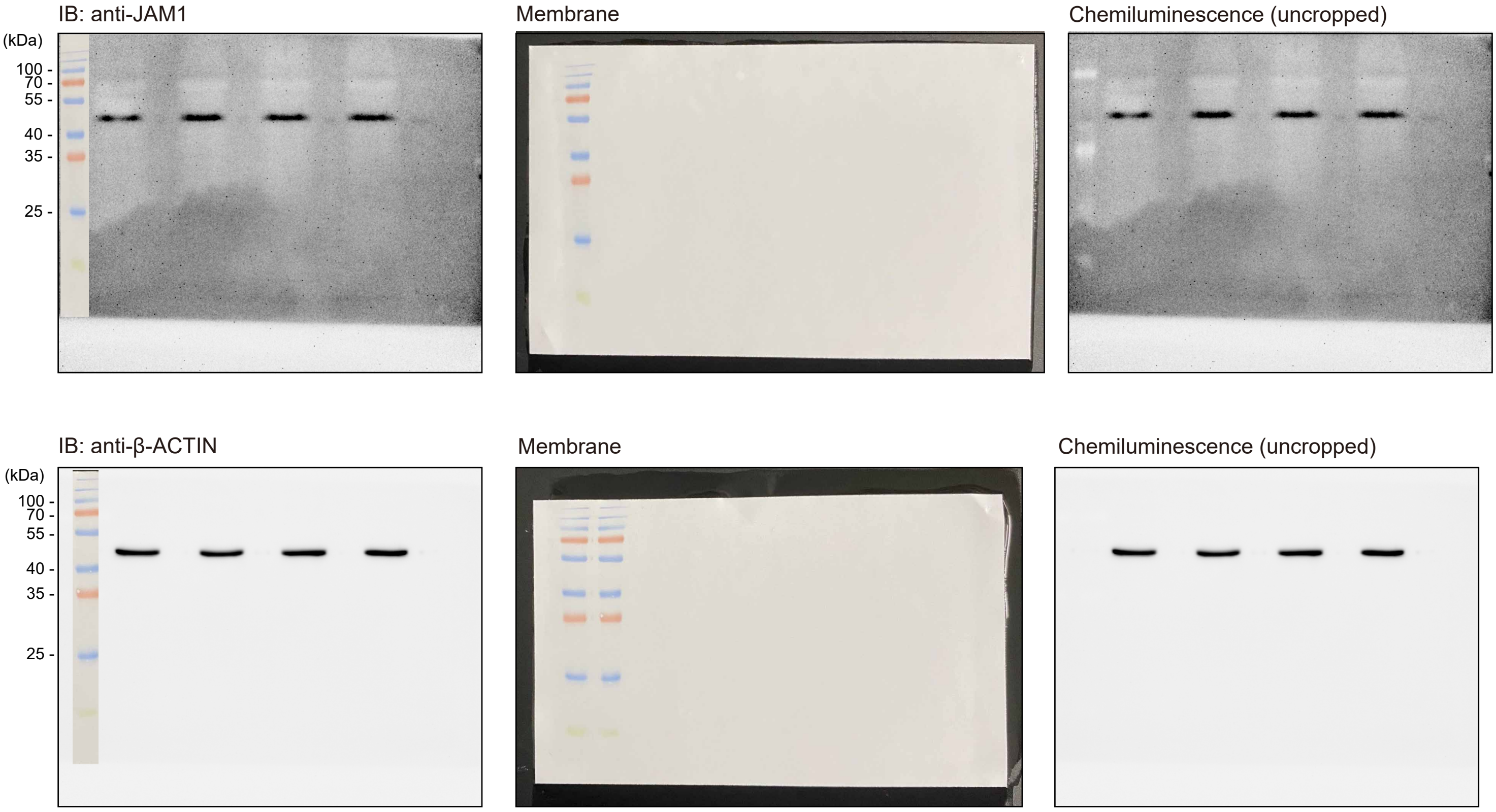

Supplementary Figure 12

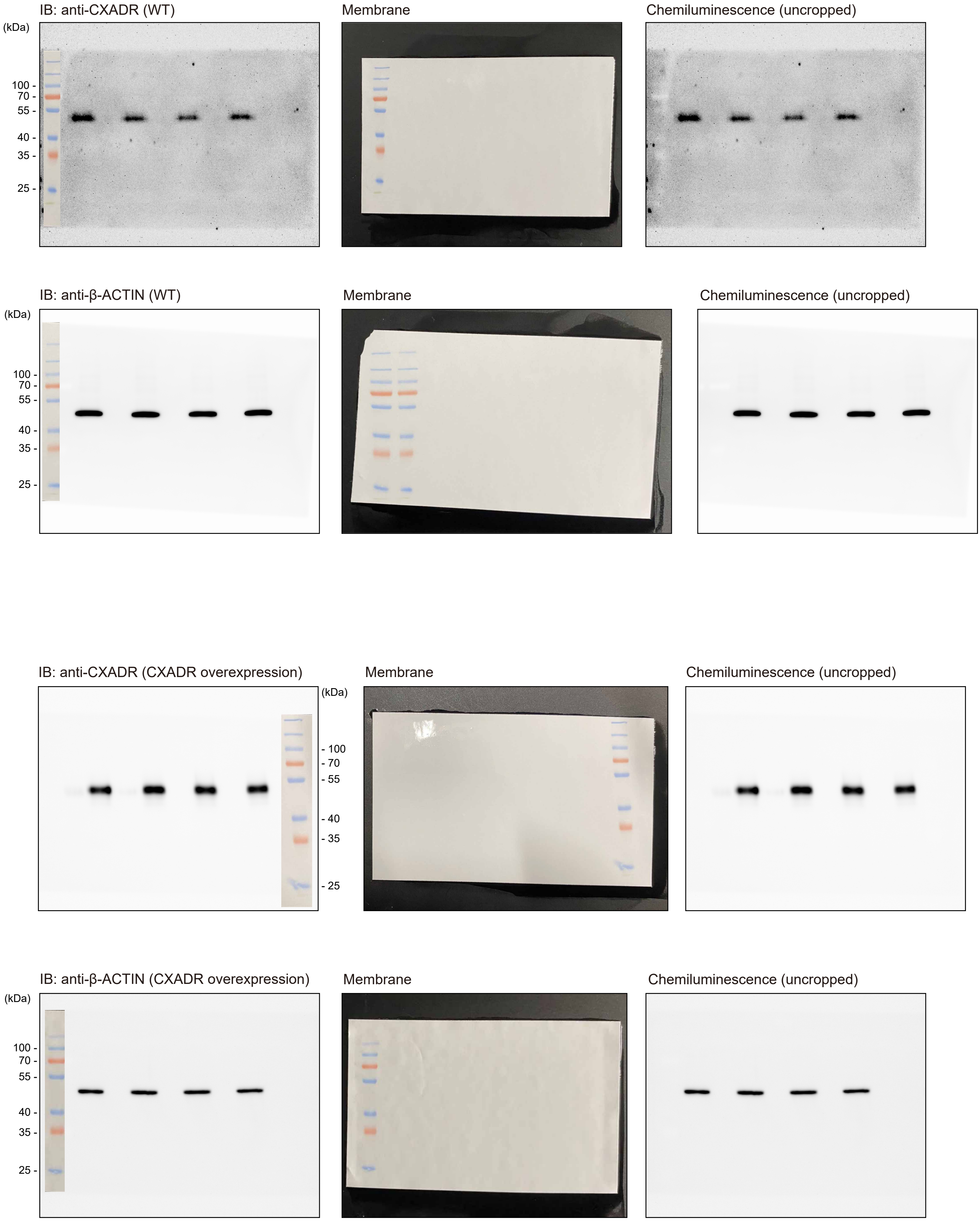

Supplement: Supplementary file 1 [file mmc1.pdf]
